# Metabotropic glutamate receptor internalization and synaptic AMPA receptor endocytosis by dopamine

**DOI:** 10.1242/jcs.264725

**Published:** 2026-08-13

**Authors:** K Aruna, Mitra Kulkarni, Samarjit Bhattacharyya

**Affiliations:** Department of Biological Sciences, Indian Institute of Science Education and Research Mohali, Mohali, Knowledge city, Sector – 81, SAS Nagar, 140306, Punjab, India

**Keywords:** Dopamine, G protein-coupled receptor, Metabotropic glutamate receptor, Receptor internalization, Synaptic plasticity

## Abstract

Group I metabotropic glutamate receptors (mGluRs) play important roles in synaptic function and plasticity, and in many neuropsychiatric disorders. Trafficking plays crucial roles in proper targeting of these receptors in specific regions of the neuron and in regulating the activity of these receptors. Group I mGluRs encounter dopamine in various regions of the brain and multiple findings suggest that dopamine regulates these receptors in the hippocampus. We show here that dopamine induces internalization of both members of the group I mGluR family, mGluR1 (also known as GRM1) and mGluR5 (GRM5), in hippocampal neurons derived from mice through a clathrin-dependent but dynamin-independent pathway. Dopamine increases mGluR-mediated Gα_q_ (also known as GNAQ) activity and phosphorylation of MAP kinases. Following dopamine-mediated internalization, receptors recycle to the cell surface faster than the recycling of agonist-mediated internalized receptors. Recycling of the dopamine-mediated internalized mGluR1 is protein phosphatase 2B dependent. Finally, we report that dopamine also induces mGluR-mediated synaptic AMPA receptor endocytosis, the cellular correlate of mGluR-dependent synaptic plasticity. Thus, this study unravels a novel role for dopamine in the internalization of mGluRs and mGluR-mediated AMPA receptor endocytosis.

## Teaser

**Summary:** Dopamine modulates the trafficking of group I metabotropic glutamate receptors (mGluRs) in hippocampal neurons and induces mGluR-mediated synaptic AMPA receptor endocytosis.

## INTRODUCTION

In the central nervous system, the majority of excitatory synapses involve glutamate as the major excitatory neurotransmitter that acts through ionotropic and metabotropic glutamate receptors (mGluRs) (Bhattacharyya, 2016). Two members of the group I mGluR family, mGluR1 (also known as GRM1) and mGluR5 (GRM5), have been implicated in various forms of experience-dependent synaptic plasticity and in several neuropsychiatric disorders, including autism and fragile X syndrome (Citri and Malenka, 2008; Dogra and Conn, 2021; Dölen et al., 2007; Su et al., 2022). Normal signaling depends on the precise location of these receptors in specific regions of the neuron and receptor trafficking plays a crucial role in controlling this localization. Furthermore, agonist-mediated intracellular trafficking can also regulate the desensitization, resensitization and intracellular signaling of these receptors (Abreu et al., 2021; Bhattacharyya, 2016; Dhami and Ferguson, 2006). In the last few years, agonist-mediated trafficking of group I mGluRs and its physiological implications have been studied in some detail.

Accumulating evidence suggests that dopamine plays an important role in hippocampus-dependent learning and memory (Fuchsberger et al., 2025; Lisman and Otmakhova, 2001; Manara et al., 2025). The hippocampus receives dopamine projections from the ventral tegmental area and *substantia nigra pars compacta* (Adeyelu and Ogundele, 2023; Gasbarri et al., 1994a,b; Sayegh et al., 2024). Importantly, specific lesion of these projections impairs spatial learning in rats (Gasbarri et al., 1996). In functional magnetic resonance imaging (fMRI) studies on humans, an association has been found between memory formation and the coactivation of midbrain dopamine areas and the hippocampus (Adcock et al., 2006; Wittmann et al., 2005). All these studies indicate a potential crosstalk between the dopaminergic system and the glutamatergic system in the hippocampus. Crosstalk between different neurotransmitter systems in the brain is not very uncommon. For example, although dopamine is not the cognate ligand for metabotropic serotonin receptors, it has been shown to directly modulate the 5-HT_1A_ (also known as HTR1A), 5-HT_2A_ (HTR2A) and 5-HT_2C_ (HTR2C) receptors (Oz et al., 2003; Woodward et al., 1992). To date, there have been no studies on the effects of dopamine on group I mGluRs, which is important to understand the potential for direct crosstalk between dopaminergic and glutamatergic systems. As dopamine-mediated mGluR trafficking as well as the effect of dopamine in mGluR-mediated AMPA receptor (AMPAR) endocytosis might have significant physiological and therapeutic implications, a better understanding of the mechanisms underlying this phenomenon is critical to establish potential effects of dopamine on the regulation of group I mGluRs.

We therefore investigated the role of dopamine in group I mGluR trafficking and mGluR-mediated AMPAR endocytosis in primary hippocampal neurons. We show here that although dopamine is not the cognate ligand for group I mGluRs, it could induce internalization of both members of the group I mGluR family, mGluR1 and mGluR5. Non-competitive antagonists of mGluR1 or mGluR5 inhibited the dopamine-mediated internalization of respective receptors. Dopamine-mediated internalization of mGluR1 was clathrin dependent, but it did not depend on dynamin. Similar to treatment with the agonist (*RS*)-3,5-dihydroxyphenylglycine (or RS-DHPG; hereafter DHPG), application of dopamine led to increase in group I mGluR-mediated Gα_q_ (also known as GNAQ) activity and increase in the phosphorylation of MAP kinases. This increase was inhibited in the presence of the non-competitive antagonists of group I mGluRs and inhibition of Gα_q_ activity. Subsequent to dopamine-mediated internalization, receptors entered the recycling compartment and recycled back to the cell surface much faster than what was observed when the receptors internalized through activation by the agonist. Furthermore, unlike the agonist-induced internalized receptors, recycling of the dopamine-mediated internalized mGluR1 was protein phosphatase 2B (PP2B) dependent and did not depend on protein phosphatase 2A (PP2A). Finally, we show that dopamine induced group I mGluR-mediated synaptic AMPAR endocytosis – the cellular correlate of mGluR-dependent synaptic plasticity. Non-competitive antagonists of group I mGluRs inhibited this process. Thus, our study unravels a completely uncharacterized role for dopamine in the internalization of group I mGluRs and group I mGluR-mediated AMPAR endocytosis, with anticipated clinical relevance to the function of these receptors in neuropsychiatric disorders.

## RESULTS

### Internalization of group I mGluRs by dopamine

Similar to many other G protein-coupled receptors (GPCRs), trafficking of group I mGluRs plays critical roles in targeting these receptors to appropriate subsynaptic and synaptic sites and controlling the activity of these receptors, both of which contribute to proper signaling by the receptor (Bhattacharyya, 2016). Both members of the group I mGluR family, namely, mGluR1 and mGluR5, also undergo rapid internalization following exposure to the agonist DHPG (Gulia et al., 2017; Iacovelli et al., 2003; Mahato et al., 2015; Ojha et al., 2022; Pandey et al., 2014; Ramsakha et al., 2023). Various reports have suggested that the neurotransmitter dopamine plays a critical role in hippocampus-dependent learning and memory, and specific lesion of dopaminergic projections inhibits spatial learning in rats (Fuchsberger et al., 2025; Gasbarri et al., 1996; Lisman and Otmakhova, 2001; Manara et al., 2025). Interestingly, an association has been observed between coactivation of midbrain dopamine areas and the hippocampal memory formation in fMRI studies on humans (Adcock et al., 2006; Wittmann et al., 2005). To investigate whether dopamine plays a role in the trafficking of group I mGluRs, we initially performed our study in heterologous HEK293 cells as these cells do not express mGluRs or dopamine receptors (Atwood et al., 2011). Moreover, HEK293 cells are one of the most widely used cell lines for heterologous expression and study of GPCRs in isolation (Thomas and Smart, 2005). HEK293 cells were transfected with a myc–mGluR1 construct and live cells expressing myc–mGluR1 were stained with anti-myc primary antibody. Subsequently, application of either 100 μM DHPG or 100 nM dopamine led to the internalization of myc–mGluR1 at 30 min (endocytosis index: control, 1.0±0.33; DHPG, 1.74±0.4; dopamine, 1.76±0.45; values show mean±s.d.) (Fig. 1A,B). We next investigated the role of dopamine in the internalization of mGluR1 in mouse primary hippocampal neurons by transfecting the neurons with the myc–mGluR1 construct and staining the live cells with anti-myc primary antibody. Similar to HEK293 cells, the receptor internalized upon application of either 100 μM DHPG or 100 nM dopamine at 30 min in primary hippocampal neurons (endocytosis index: control, 1.0±0.35; DHPG, 1.92±0.39; dopamine, 1.93±0.46) (Fig. 1C,D). We chose 30 min as the time point as our previous studies suggested that DHPG-mediated internalization of myc–mGluR1 and myc–mGluR5 reached the maximum level at 30 min post ligand application (Mahato et al., 2015; Pandey et al., 2014). In order to rule out the possibility that dopamine-mediated internalization of myc–mGluR1 was due to the activation of dopamine receptors by dopamine, we performed the endocytosis experiments in the presence of dopamine receptor blockers (10 μM chlorpromazine hydrochloride and 10 μM haloperidol). At these concentrations, chlorpromazine hydrochloride and haloperidol inhibit both D1-like (D1 and D5 subtypes) and D2-like (D2, D3 and D4 subtypes) dopamine receptors (Faedda et al., 1989). myc–mGluR1-expressing primary hippocampal neurons were pre-treated with dopamine receptor blockers for 30 min before application of dopamine and the blockers were present until the end of the experiment. In both control cells and cells treated with the dopamine receptor blockers, application of 100 nM dopamine led to the internalization of myc–mGluR1 (endocytosis index: control, 1.0±0.29; dopamine, 2.09±0.61; dopamine+blockers, 2.12±0.5) (Fig. 1E,F). However, application of dopamine receptor blockers alone did not cause any endocytosis of myc–mGluR1 (Fig. S1A,B). Interestingly, treatment with the non-competitive antagonist of mGluR1, JNJ16259685 (30 μM), inhibited the dopamine-mediated internalization of myc–mGluR1 (endocytosis index: control, 1.0±0.23; dopamine, 2.16±0.45; dopamine+JNJ16259685, 1.02±0.2) (Fig. 1G,H).

**Fig. 1.**
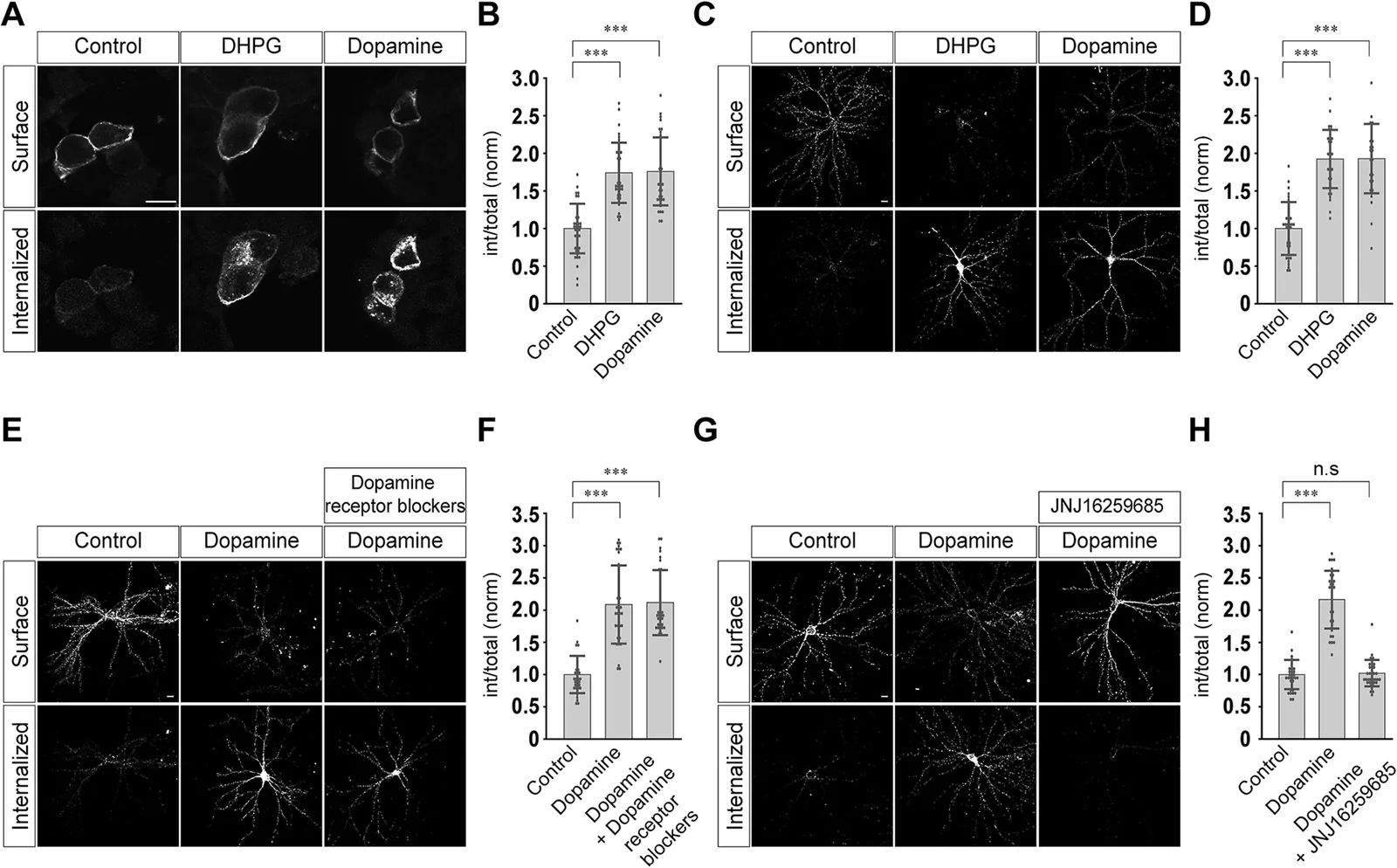
**Dopamine induces internalization of mGluR1 in HEK293 cells and primary hippocampal neurons.** (A,B) Representative images (A) and quantification of the endocytosis index (B) suggested that both 100 μM DHPG and 100 nM dopamine induced internalization of myc–mGluR1 in HEK293 cells (*P*=2.417×10^−13^ between control and DHPG; *P*=1.251×10^−12^ between control and dopamine; one-way ANOVA followed by Tukey's post-test). (C,D) Representative images (C) of surface and internalized myc–mGluR1, 30 min post application of 100 μM DHPG or 100 nM dopamine in primary hippocampal neurons, and quantification of the endocytosis index (D) suggested that both DHPG and dopamine induced the internalization of myc–mGluR1 in neurons (*P*=2.358×10^−15^ between control and DHPG; *P*=3.085×10^−13^ between control and dopamine; one-way ANOVA followed by Tukey's post-test). (E,F) Representative cells (E) and quantification (F) of 100 nM dopamine-induced internalization of myc–mGluR1 in the absence and presence of the dopamine receptor blockers suggested that dopamine induced internalization of myc–mGluR1 in both conditions in the primary hippocampal neurons (*P*=7.236×10^−14^ between control and dopamine; *P*=1.854×10^−16^ between control and dopamine+blockers; one-way ANOVA followed by Tukey's post-test). (G,H) Application of 30 μM JNJ16259685 led to the inhibition of the dopamine-mediated internalization of myc–mGluR1 in primary hippocampal neurons as shown by the representative images (G) and quantification of the endocytosis index (H) (*P*=1.535×10^−19^ between control and dopamine; *P*=0.682 between control and dopamine+JNJ16259685; one-way ANOVA followed by Tukey's post-test). Results are presented as mean±s.d. collected from three independent experiments. norm, normalized. Scale bars: 10 μm. n.s, not significant, *P*>0.05; ***P<0.001.

We subsequently investigated the role of dopamine, if any, in the endocytosis of the other member of the group I mGluR family, namely, mGluR5. Treatment with 100 μM DHPG and 100 nM dopamine both induced internalization of myc–mGluR5 in HEK293 cells (endocytosis index: control, 1.0±0.25; DHPG, 1.84±0.3; dopamine, 1.85±0.36) (Fig. 2A,B). Similar to HEK293 cells, application of 100 μM DHPG or 100 nM dopamine led to the internalization of myc–mGluR5 in primary hippocampal neurons as well (endocytosis index: control, 1.0±0.15; DHPG, 1.93±0.28; dopamine, 1.97±0.25) (Fig. 2C,D). Furthermore, 100 nM dopamine induced the internalization of myc–mGluR5 in the presence of the dopamine receptor blockers in primary hippocampal neurons (endocytosis index: control, 1.0±0.24; dopamine, 1.9±0.35; dopamine+blockers, 1.97±0.2) (Fig. 2E,F). Similar to what was observed in the case of myc–mGluR1, application of dopamine receptor blockers alone did not induce internalization of myc–mGluR5 (Fig. S1C,D). Importantly, treatment with a non-competitive antagonist of mGluR5, 3-[(2-methyl-4-thiazolyl) ethynyl] pyridine (MTEP; 100 μM), blocked the dopamine-mediated internalization of myc–mGluR5 (endocytosis index: control, 1.0±0.21; dopamine, 1.74±0.32; dopamine+MTEP, 1.04±0.18) (Fig. 2G,H). Thus, these results suggest that, similar to the agonist DHPG, dopamine can also induce internalization of both mGluR1 and mGluR5 in heterologous HEK293 cells as well as in primary hippocampal neurons. Furthermore, the non-competitive antagonist of mGluR1 and mGluR5 inhibits the dopamine-mediated internalization of respective receptors. As both mGluR1 and mGluR5 showed similar results upon application of dopamine, we concentrated on mGluR1 for the rest of the study.

**Fig. 2.**
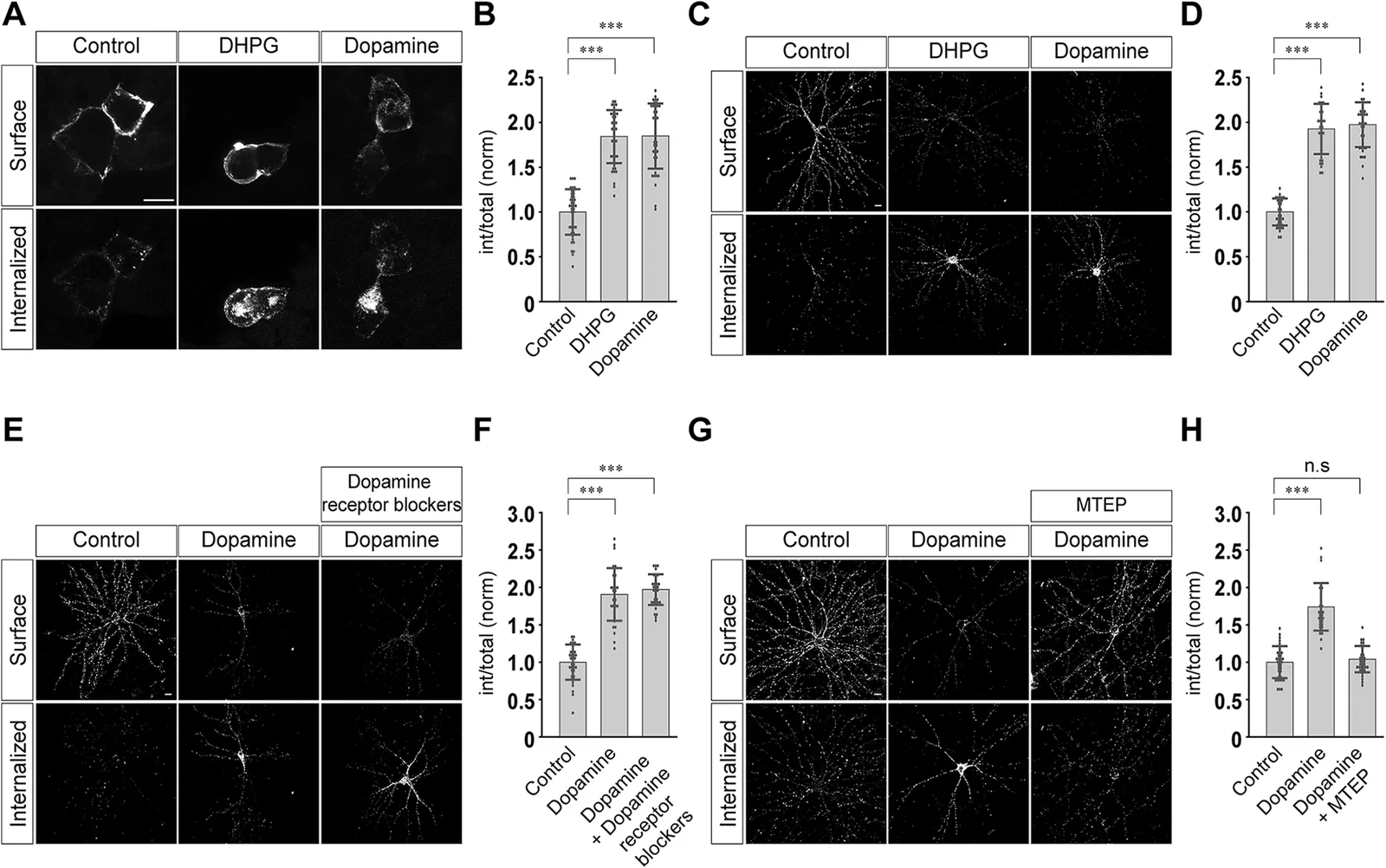
**Internalization of mGluR5 in HEK293 cells and primary hippocampal neurons by dopamine.** (A,B) Representative images (A) and quantification of the internalization index (B) shows that application of 100 μM DHPG and 100 nM dopamine led to the internalization of myc–mGluR5 in HEK293 cells (*P*=9.114×10^−24^ between control and DHPG; *P*=2.203×10^−20^ between control and dopamine; one-way ANOVA followed by Tukey's post-test). (C,D) Representative images (C) and quantification of the endocytosis index (D) suggested that both 100 μM DHPG and 100 nM dopamine induced the internalization of myc–mGluR5 in primary hippocampal neurons (*P*=1.582×10^−25^ between control and DHPG; *P*=2.251×10^−29^ between control and dopamine; one-way ANOVA followed by Tukey's post-test). (E,F) 100 nM dopamine induced internalization of myc–mGluR5 in the absence and presence of the dopamine receptor blockers in the primary hippocampal neurons as shown by the representative images (E) and quantification of the endocytosis index (F) (*P*=1.179×10^−19^ between control and dopamine; *P*=3.201×10^−27^ between control and dopamine+blockers; one-way ANOVA followed by Tukey's post-test). (G,H) Representative images (G) and quantification of the internalization index (H) suggested that application of 100 μM MTEP resulted in the inhibition of the dopamine-mediated internalization of myc–mGluR5 in primary hippocampal neurons (*P*=2.979×10^−16^ between control and dopamine; *P*=0.407 between control and dopamine+MTEP; one-way ANOVA followed by Tukey's post-test). Results are presented as mean±s.d. collected from three independent experiments. norm, normalized. Scale bars: 10 μm. n.s, not significant, *P*>0.05; ***P<0.001.

### Dopamine-mediated internalization of mGluR1 is clathrin dependent and dynamin independent

GPCRs endocytose by multiple different pathways (Drake et al., 2006; Martinez-Morales et al., 2022; Sposini and Hanyaloglu, 2018). What pathway a particular receptor takes depends on the type of the ligand, the type of the receptor and the cellular background. It has been reported that agonist-mediated internalization of mGluR1 is arrestin and dynamin dependent (Mundell et al., 2001). In contrast, the constitutive endocytosis of mGluR5 has been reported to be clathrin independent (Fourgeaud et al., 2003). Our results suggest that 1 nM dopamine does not induce significant internalization of myc–mGluR1 in primary hippocampal neurons. However, myc–mGluR1 internalized upon application of 100 nM dopamine, and application of 10 μM dopamine did not increase the internalization of the receptor further (endocytosis index: control, 1.0±0.42; 1 nM dopamine, 1.02±0.53; 100 nM dopamine, 1.87±0.42; 10 μM dopamine, 1.85±0.42) (Fig. 3A,B). The dopamine-mediated internalization of myc–mGluR1 was observed to be rapid and an increase in the internalization of myc–mGluR1 was observed 3 min post dopamine application in primary hippocampal neurons (endocytosis index: control, 1.0±0.32; 3 min, 1.24±0.43) (Fig. 3C,D). The amount of myc–mGluR1 internalization increased over time (5 min, 1.58±0.47; 30 min, 1.67±0.52) (Fig. 3C,D). In order to investigate whether the dopamine-mediated internalization of mGluR1 is clathrin and dynamin dependent, we studied the dopamine-mediated internalization of myc–mGluR1 by pharmacologically inhibiting clathrin and dynamin in primary hippocampal neurons. myc–mGluR1 expressing primary hippocampal neurons were pre-treated with either 30 μM pitstop 2 or 80 μM dynasore for 30 min to inhibit endogenous clathrin and dynamin, respectively. In control cells, myc–mGluR1 internalized upon application of 100 nM dopamine (endocytosis index: control, 1.0±0.21; control+dopamine, 2.06±0.45) (Fig. 4A,B). Inhibition of clathrin by pitstop 2 resulted in the inhibition of the dopamine-mediated internalization of myc–mGluR1, whereas inhibition of dynamin with dynasore had no effect on the internalization of the receptor (endocytosis index: pitstop 2+dopamine, 0.98±0.25; dynasore+dopamine, 1.91±0.47) (Fig. 4A,B). In order to negate the possibility that dopamine-mediated internalization of myc–mGluR1 was not inhibited by the application of dynasore due to the non-activity of dynasore in our experiments, we studied the DHPG-mediated internalization of myc–mGluR1 in the presence of dynasore. As has been mentioned earlier, the agonist-mediated internalization of mGluR1 has been reported to be dynamin-dependent (Mundell et al., 2001). As expected, application of 80 μM dynasore inhibited the 100 μM DHPG-mediated internalization of myc–mGluR1 in primary hippocampal neurons, suggesting that the dopamine-mediated internalization of mGluR1 is indeed dynamin independent (Fig. S2A,B).

**Fig. 3.**
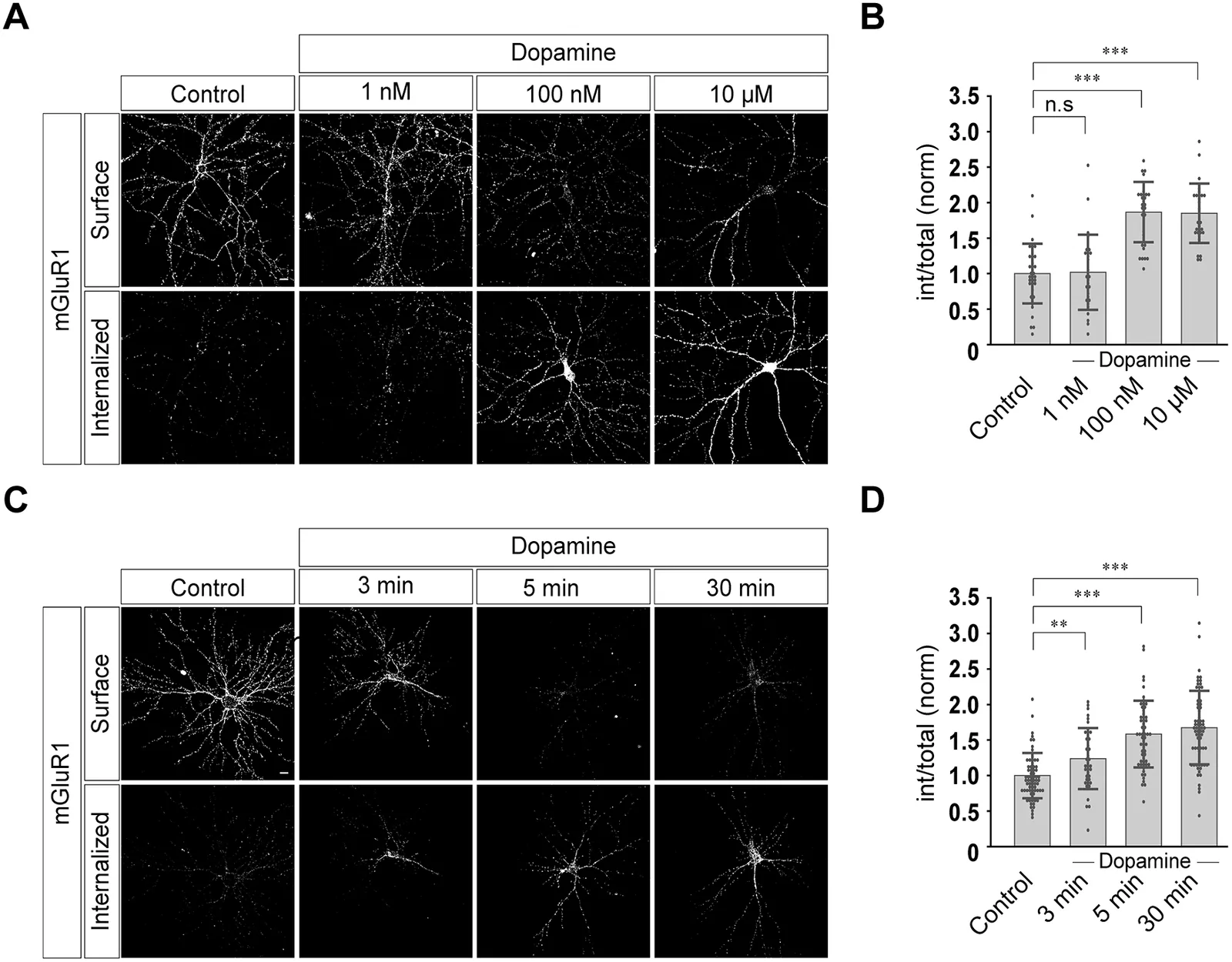
**Dose response and time course of dopamine-mediated internalization of mGluR1.** (A,B) Representative images (A) and quantification of the endocytosis index (B) showing that application of 1 nM dopamine did not induce internalization of myc–mGluR1 in primary hippocampal neurons. Maximum internalization of the receptor was observed upon application of 100 nM dopamine and 10 μM dopamine did not increase the internalization of myc–mGluR1 further (*P*=0.869 between control and 1 nM dopamine; *P*=1.858×10^−11^ between control and 100 nM dopamine; *P*=4.182×10^−11^ between control and 10 μM dopamine; one-way ANOVA followed by Tukey's post-test). (C,D) The kinetics of dopamine-mediated internalization of myc–mGluR1 in primary hippocampal neurons suggested that internalization of myc–mGluR1 progressively increased until 30 min upon application of 100 nM dopamine as shown by the representative images (C) and quantification of the endocytosis index (D) (*P*=0.002 between control and 3 min; *P*=1.421×10^−13^ between control and 5 min; *P*=4.54×10^−16^ between control and 30 min; one-way ANOVA followed by Tukey's post-test). All results are presented as mean±s.d. collected from three independent experiments. norm, normalized. Scale bars: 10 μm. n.s, not significant, *P*>0.05; **P<0.01; ***P<0.001.

**Fig. 4.**
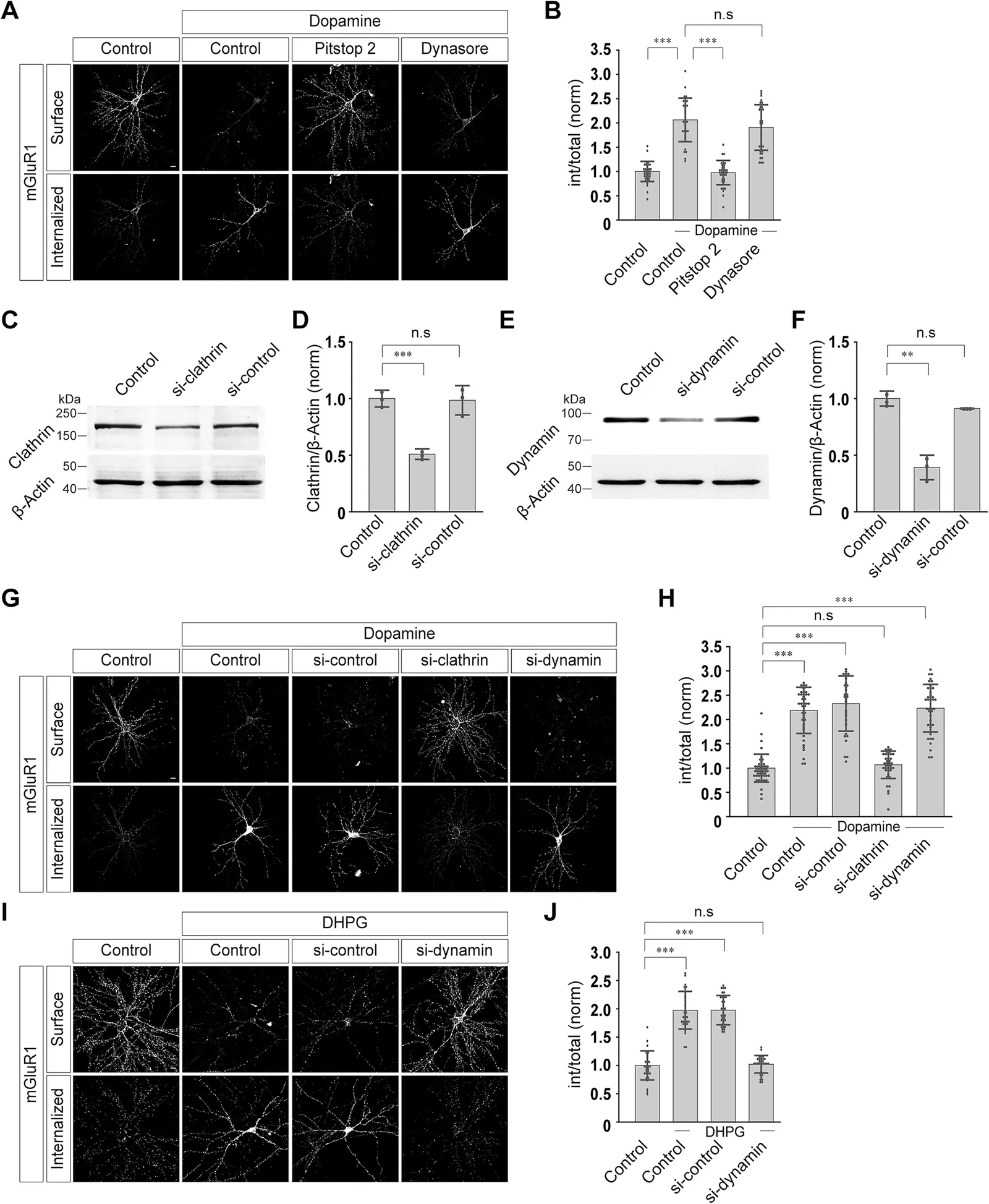
**Dopamine-mediated internalization of mGluR1 is clathrin dependent and dynamin independent.** (A,B) Dopamine-mediated internalization of myc–mGluR1 was inhibited upon inhibition of clathrin by pitstop 2, but inhibition of dynamin by dynasore had no effect on the internalization of the receptor as shown by the representative images (A) and quantification of the endocytosis index (B) (*P*=2.651×10^−19^ between control and control+dopamine; *P*=1.407×10^−18^ between control+dopamine and pitstop 2+dopamine; *P*=0.169 between control+dopamine and dynasore+dopamine; one-way ANOVA followed by Tukey's post-test). (C,D) Western blot analyses (C) and quantification of the western blots (D) showing the efficient knockdown of endogenous clathrin by si-clathrin (*P*=6.507×10^−4^ between control and si-clathrin; *P*=0.876 between control and si-control; one-way ANOVA followed by Tukey's post-test). (E,F) Acute knockdown of the endogenous dynamin by si-dynamin, as shown by the western blots (E) and quantification of the western blots (F) (*P*=0.001 between control and si-dynamin; *P*=0.082 between control and si-control; one-way ANOVA followed by Tukey's post-test). (G,H) Representative images (G) and quantification of the endocytosis index (H) suggested that knockdown of endogenous clathrin resulted in the inhibition of the dopamine-mediated internalization of myc–mGluR1, whereas in control cells, si-control-treated cells and si-dynamin-transfected cells, the receptor showed normal internalization upon 100 nM dopamine application (*P*=7.152×10^−25^ between control and control+dopamine; *P*=3.982×10^−22^ between control and si-control+dopamine; *P*=0.274 between control and si-clathrin+dopamine; *P*=3.629×10^−25^ between control and si-dynamin+dopamine; one-way ANOVA followed by Tukey's post-test). (I,J) Knockdown of the endogenous dynamin inhibited the DHPG-mediated internalization of myc–mGluR1, whereas in control cells and si-control transfected cells normal internalization of the receptor was observed, as shown by the representative images (I) and quantification of the endocytosis index (J) (*P*=1.391×10^−18^ between control and control+DHPG; *P*=9.533×10^−23^ between control and si-control+DHPG; *P*=0.709 between control and si-dynamin+DHPG; one-way ANOVA followed by Tukey's post-test). All results are presented as mean±s.d. collected from three independent experiments. norm, normalized. Scale bars: 10 μm. n.s, not significant, *P*>0.05; **P<0.01; ***P<0.001.

We subsequently studied the role of clathrin and dynamin in the dopamine-mediated internalization of myc–mGluR1 by acute knockdown of endogenous clathrin and dynamin in primary hippocampal neurons. We knocked down dynamin-1 (DNM1, hereafter referred to as dynamin) as this isoform of dynamin is almost exclusively expressed in the central nervous system (Ferguson et al., 2007; Sontag et al., 1994). Cells were co-transfected with the myc–mGluR1 construct and siRNA against either endogenous clathrin heavy chain (*Cltc*; si-clathrin) or endogenous dynamin (*Dnm1*; si-dynamin). The efficiency of knockdown of endogenous clathrin and dynamin was checked by western blot analyses 72 h post transfection as described in the Materials and Methods. Our data suggested that si-clathrin efficiently knocked down endogenous clathrin in primary neurons (normalized protein levels: control, 1.0±0.07; si-clathrin, 0.51±0.05; si-control, 0.99±0.13) (Fig. 4C,D). Similarly, si-dynamin also knocked down expression of endogenous dynamin efficiently (normalized protein levels: control, 1.0±0.07; si-dynamin, 0.39±0.11; si-control, 0.91±0.003) (Fig. 4E,F). Importantly, knockdown of endogenous clathrin led to the complete inhibition of the dopamine-mediated internalization of myc–mGluR1, whereas knockdown of endogenous dynamin had no effect on the dopamine-mediated internalization of the receptor (endocytosis index: control, 1.0±0.29; control+dopamine, 2.19±0.47; si-control+dopamine, 2.33±0.57; si-clathrin+dopamine, 1.07±0.28; si-dynamin+dopamine, 2.23±0.49) (Fig. 4G,H). We further investigated the role of clathrin in the dopamine-mediated internalization of myc–mGluR1 by acute knockdown of endogenous clathrin using shRNA. We initially cloned a shRNA against clathrin (sh-clathrin) in a multipromoter vector under the H1 promoter as described in the Materials and Methods. It has been reported that this shRNA effectively knocked down endogenous clathrin in primary hippocampal neurons (Watanabe et al., 2014). Primary hippocampal neurons were co-transfected with myc–mGluR1 and sh-clathrin or sh-control constructs. Live cells expressing myc–mGluR1 were stained with anti-myc primary antibody. Subsequent application of 100 nM dopamine led to the internalization of myc–mGluR1 in control cells and in sh-control-transfected cells. However, cells expressing sh-clathrin did not show significantly internalized receptors upon 100 nM dopamine application and most of the receptors were observed to be localized at the cell surface (Fig. S3A,B). As mentioned before, agonist-mediated internalization of mGluR1 is dynamin dependent (Mundell et al., 2001). In order to rule out the possibility that knockdown of endogenous dynamin did not affect the dopamine-mediated internalization of the receptor due to the larger reserve of dynamin than clathrin in the cell subsequent to the knockdown of the endogenous protein, we studied the agonist-mediated internalization of myc–mGluR1 upon acute knockdown of endogenous dynamin. Knockdown of endogenous dynamin led to the complete inhibition of the 100 μM DHPG-mediated internalization of myc–mGluR1, whereas control cells and si-control-transfected cells showed normal endocytosis of the receptor (endocytosis index: control, 1.0±0.26; control+DHPG, 1.97±0.33; si-control+DHPG, 1.98±0.26; si-dynamin+DHPG, 1.02±0.16) (Fig. 4I,J). These results suggest that dopamine-mediated internalization of mGluR1 is clathrin dependent but dynamin independent.

### Group I mGluRs activate the Gα_q_-mediated signal transduction pathway upon dopamine application

Group I mGluR activation by the endogenous ligand glutamate or the agonist DHPG triggers G protein activity and downstream signal transduction pathways. Both mGluR1 and mGluR5 are Gα_q_-linked GPCRs, which activate phospholipase C to generate diacylglycerol and inositol 1,4,5-triphosphate (Conn and Pin, 1997; Kim et al., 2008). Group I mGluRs also upregulate the phosphorylation of the MAP kinases ERK1 (also known as MAPK3) and ERK2 (also known as MAPK1) (hereafter ERK1/2) upon activation by the agonist (Gladding et al., 2009; Kim et al., 2008; Ojha et al., 2022; Sharma et al., 2018). In order to investigate whether application of dopamine leads to the activation of group I mGluRs and G protein activity, we first studied the interaction of mGluR1 with Gα_q_ before and after the application of dopamine. HEK293 cells were transfected with empty vector or a FLAG–mGluR1 construct. Subsequently, co-immunoprecipitation experiments were performed in the absence or presence of DHPG and dopamine. Our results suggested that, as expected, application of 100 μM DHPG resulted in the increased interaction of FLAG–mGluR1 with Gα_q_ compared to the control condition. Interestingly, we observed an increased interaction of FLAG–mGluR1 with Gα_q_ subsequent to the application of 100 nM dopamine as well (normalized Gα_q_/mGluR1 ratio: control, 1.0±0.43; DHPG, 3.09±0.52; dopamine, 3.01±0.41) (Fig. 5A,B). Importantly, the magnitude of increased interaction between FLAG–mGluR1 and Gα_q_ on dopamine application was very similar to that was observed when 100 μM DHPG was applied.

**Fig. 5.**
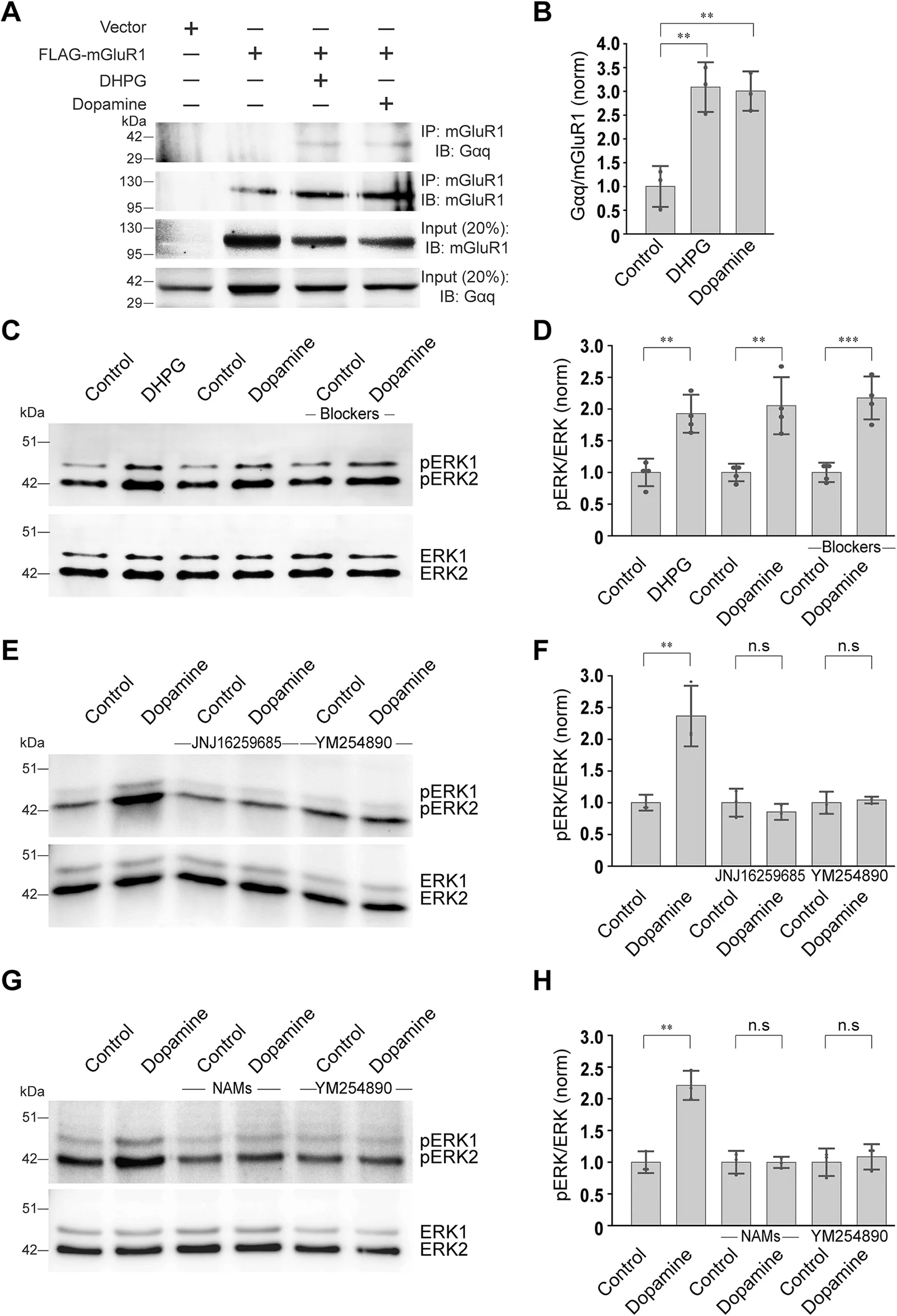
**Group I mGluRs activate Gα_q_-mediated second messenger pathway following dopamine application.** (A,B) Co-immunoprecipitation assay (A) and quantification of the co-immunoprecipitation assays (B) suggesting that application of both 100 μM DHPG and 100 nM dopamine increased the interaction of Gα_q_ with FLAG–mGluR1 (*P*=0.006 between control and DHPG; *P*=0.004 between control and dopamine; one-way ANOVA followed by Tukey's post-test). IB, immunoblotting; IP, immunoprecipitate. (C,D) Western blot (C) and quantification of western blots (D) showing that application of 100 μM DHPG and 100 nM dopamine increased the phosphorylation of the MAP kinases ERK1 and ERK2 compared to that in control cells. 100 nM dopamine also increased the phosphorylation of MAP kinases in the presence of dopamine receptor blockers (*P*=0.002 between control and DHPG; *P*=0.004 between control and dopamine; *P*=7.345×10^−4^ between control+blockers and dopamine+blockers; one-way ANOVA followed by Tukey's post-test). (E,F) In HEK293 cells, application of 100 nM dopamine led to an increase in the phosphorylation of MAP kinases compared to that in control cells. The non-competitive inhibitor of mGluR1, JNJ16259685, and the Gα_q_ inhibitor YM254890 inhibited this dopamine-mediated increase in the phosphorylation of MAP kinases as shown in the western blot (E) and quantification of western blots (F) (*P*=0.009 between control and dopamine; *P*=0.378 between control+JNJ16259685 and dopamine+JNJ16259685; *P*=0.714 between control+YM254890 and dopamine+YM254890; one-way ANOVA followed by Tukey's post-test). (G,H) 100 nM dopamine increased the phosphorylation of MAP kinases in primary neurons compared to that in control cells. Application of non-competitive antagonists (negative allosteric modulators or NAMs) and the Gα_q_ blocker YM254890 both inhibited the 100 nM dopamine-mediated increase of the phosphorylation of MAP kinases as shown in western blots (G) and quantification of western blots (H) (*P*=0.002 between control and dopamine; *P*=0.984 between control+NAMs and dopamine+NAMs; *P*=0.643 between control+YM254890 and dopamine+YM254890; one-way ANOVA followed by Tukey's post-test). All results are presented as mean±s.d. collected from three independent experiments. norm, normalized. n.s, not significant, *P*>0.05; **P<0.01; ***P<0.001.

As mentioned before, group I mGluRs upregulate the phosphorylation of ERK1/2 on activation by the agonist. To investigate whether group I mGluRs upregulate the phosphorylation of ERK1/2 upon application of dopamine, primary hippocampal neurons were treated with DHPG and dopamine. In control cells, application of 100 μM DHPG increased the phosphorylation of ERK1/2 [normalized phosphorylated (p)ERK/ERK ratio: control, 1.0±0.22; DHPG, 1.93±0.3] (Fig. 5C,D). Application of 100 nM dopamine also increased the phosphorylation of ERK1/2 (normalized pERK/ERK ratio: control, 1.0±0.14; dopamine, 2.05±0.45) (Fig. 5C,D). In order to rule out the possibility that dopamine-mediated increase in the phosphorylation of ERK1/2 was due to the activation of dopamine receptors by dopamine, we performed the same experiments in presence of the dopamine receptor blockers. 100 nM dopamine increased the phosphorylation of ERK1/2 even in presence of the dopamine receptor blockers, suggesting that increase in the phosphorylation of ERK1/2 upon dopamine application was not due to the activation of dopamine receptors by dopamine (normalized pERK/ERK ratio: control+blockers, 1.0±0.15; dopamine+blockers, 2.17±0.34) (Fig. 5C,D).

Our previous results suggested that dopamine-mediated internalization of group I mGluRs was inhibited by their respective non-competitive antagonists. Therefore, we next investigated whether the activation of ERK1/2 pathway by dopamine also gets inhibited by the non-competitive antagonists of mGluR1 and mGluR5. In addition, we also studied whether inhibition of Gα_q_ activity leads to the inhibition of the activation of the ERK1/2 pathway by dopamine through group I mGluRs. Initially, we performed these experiments in heterologous HEK293 cells. Cells were transfected with the myc–mGluR1 construct. Subsequently, cells were either treated with the mGluR1 antagonist JNJ16259685 (30 μM) or the Gα_q_ inhibitor YM254890 (10 μM). The ability of dopamine to upregulate the phosphorylation of ERK1/2 in presence of these blockers was investigated following the protocol described in the Materials and Methods. Application of 100 nM dopamine increased the phosphorylation of ERK1/2 in control cells. Importantly, dopamine could not increase the phosphorylation of ERK1/2 in presence of the mGluR1 antagonist or the Gα_q_ inhibitor (normalized pERK/ERK ratio: control, 1.0±0.12; dopamine, 2.37±0.48; control+JNJ16259685, 1.0±0.22; dopamine+JNJ16259685, 0.86±0.12; control+YM254890, 1.0±0.18; dopamine+YM254890, 1.04±0.05) (Fig. 5E,F). Finally, we performed these experiments in primary neurons. Cells were treated with either group I mGluR non-competitive antagonists (negative allosteric modulators or NAMs; 30 μM JNJ16259685 and 100 μM MTEP) or the Gα_q_ inhibitor (10 μM YM254890). Subsequently, we investigated whether group I mGluRs upregulate the phosphorylation of ERK1/2 upon application of dopamine in the presence of these blockers using the same protocol as described before. As expected, application of 100 nM dopamine increased the phosphorylation of ERK1/2. In contrast, application of group I mGluR non-competitive antagonists or inhibition of Gα_q_ activity both inhibited the dopamine-mediated increase in the phosphorylation of ERK1/2 (normalized pERK/ERK ratio: control, 1.0±0.17; dopamine, 2.21±0.23; control+NAMs, 1.0±0.18; dopamine+NAMs, 1±0.09; control+YM254890, 1.0±0.22; dopamine+YM254890, 1.09±0.2) (Fig. 5G,H). Thus, these results suggest that, similar to the agonist DHPG, dopamine also increases Gα_q_ activity mediated by group I mGluRs and subsequent activation of the ERK1/2 pathway.

### Group I mGluRs recycle to the cell surface following dopamine-mediated internalization

Subsequent to the ligand-dependent internalization, GPCRs can have various subcellular fates. Some receptors enter the recycling compartment and recycle back to the cell surface, whereas other GPCRs enter the lysosome for degradation following endocytosis (Drake et al., 2006; Ferguson, 2001). Our earlier studies suggested that both mGluR1 and mGluR5 recycle to the cell surface subsequent to agonist-mediated internalization (Mahato et al., 2015; Pandey et al., 2014). In order to investigate the intracellular fate of mGluR1 following dopamine-mediated internalization, we studied the intracellular localization of the dopamine-mediated internalized receptors. Primary hippocampal neurons expressing myc–mGluR1 were treated with 100 nM dopamine for 5 min subsequent to anti-myc primary antibody staining and cells were chased for 25 min in the absence of dopamine. Surface stripping of the cells was done, followed by fixation and permeabilization of the cells. Cells were then stained with primary antibodies against either Rab11 (also known as RAB11A; recycling endosome marker) or LAMP1 (lysosomal marker), followed by the appropriate secondary antibodies to visualize both internalized receptors and compartment markers as described in the Materials and Methods. myc–mGluR1 showed significant increase in colocalization with Rab11 following dopamine-mediated internalization (colocalization index of internalized mGluR1 with Rab11: control, 1.0±0.49; 30 min, 2.51±0.44) (Fig. 6A,B). In contrast, no significant increase in the colocalization of the dopamine-mediated internalized myc–mGluR1 was observed with LAMP1 (colocalization index of internalized mGluR1 with LAMP1; control, 1.0±0.6; 30 min, 1±0.19) (Fig. 6C,D). These results suggest that, following dopamine-mediated internalization, myc–mGluR1 enters the Rab11-positive recycling compartment.

**Fig. 6.**
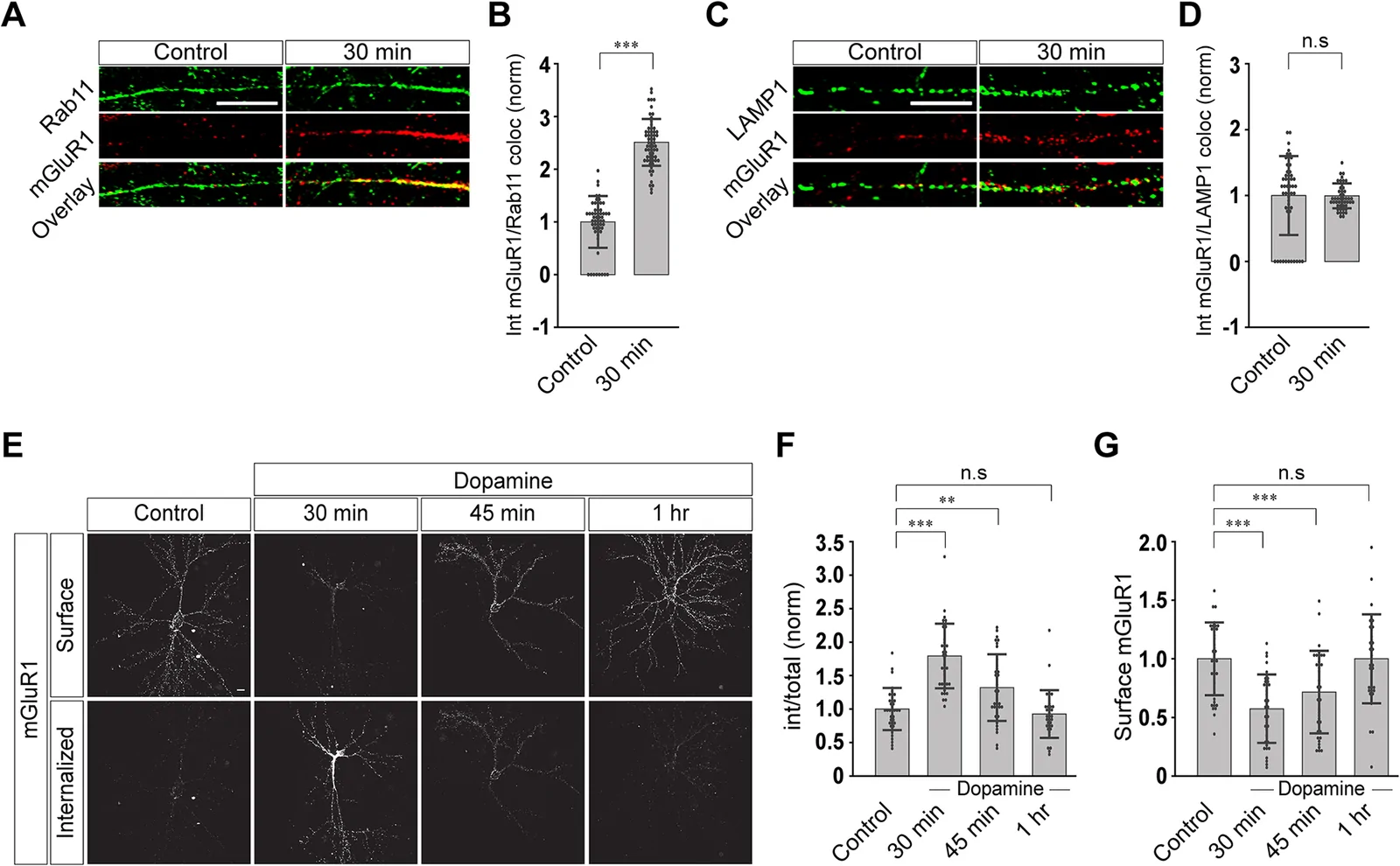
**mGluR1 recycles back to the cell surface subsequent to dopamine-mediated internalization.** (A,B) Representative images (A) and quantification (B) suggest that following dopamine-mediated internalization, myc–mGluR1 colocalized with the recycling endosome marker Rab11 (*P*=2.196×10^−34^ between control and 30 min; two-tailed unpaired *t*-test). (C,D) In contrast, following dopamine-induced endocytosis, myc–mGluR1 did not show increase in the colocalization with the lysosomal maker LAMP1 in primary hippocampal neurons as shown in representative images (C) and quantification (D) (*P*=0.965 between control and 30 min; two-tailed unpaired *t*-test). (E) Representative images showing the kinetics of dopamine-induced internalization and recycling of myc–mGluR1 in primary hippocampal neurons. (F,G) Upon application of 100 nM dopamine, internalization of myc–mGluR1 increased until 30 min and the receptor recycled back to the cell surface in 1 h as shown by the quantification of the endocytosis index (F) (*P*=4.804×10^−12^ between control and 30 min; *P*=0.002 between control and 45 min; *P*=0.36 between control and 1 h; one-way ANOVA followed by Tukey's post-test) as well as measurement of the surface myc–mGluR1 (G) (*P*=6.431×10^−8^ between control and 30 min; *P*=5.356×10^−4^ between control and 45 min; *P*=0.999 between control and 1 h; one-way ANOVA followed by Tukey's post-test). All results are presented as mean±s.d. collected from three independent experiments. norm, normalized. Scale bars: 10 μm. n.s, not significant, *P*>0.05; **P<0.01; ***P<0.001.

We then studied the recycling of myc–mGluR1 following dopamine-mediated internalization. Live primary hippocampal neurons expressing myc–mGluR1 were stained with anti-myc primary antibody. Application of 100 nM dopamine for 5 min led to the internalization of myc–mGluR1 at 30 min and, when cells were chased for longer periods in the absence of dopamine, the majority of the receptors recycled back to the cell surface in 1 h (endocytosis index: control, 1.0±0.31; 30 min, 1.79±0.48; 45 min, 1.32±0.5; 1 h, 0.93±0.36) (Fig. 6E,F). Measurement of the myc–mGluR1 fluorescence at the cell surface also showed recovery similar to control levels, 1 h post dopamine-mediated internalization of the receptor (normalized surface mGluR1 levels: control, 1.0±0.31; 30 min, 0.57±0.29; 45 min, 0.72±0.35; 1 h, 1±0.38) (Fig. 6E,G). We reported earlier that myc–mGluR1 normally recycles back to the cell surface at 2.5 h after agonist-mediated internalization (Pandey et al., 2014). Thus, these results suggest that myc–mGluR1 undergoes faster recycling following dopamine-mediated internalization. myc–mGluR5 also recycled back to the cell surface in 1 h subsequent to the dopamine-mediated internalization in primary hippocampal neurons (Fig. S4A–C). We have shown earlier that myc–mGluR5 recycles back to the cell surface in 3 h following agonist-mediated internalization (Mahato et al., 2015). Therefore, these results suggest that, similar to myc–mGluR1, myc–mGluR5 also undergoes faster recycling subsequent to the dopamine-mediated internalization.

### Recycling of mGluR1 following dopamine-mediated internalization is PP2B dependent

Our earlier studies suggested that recycling of mGluR1 subsequent to the agonist-mediated internalization is dependent on the activity of PP2A and does not depend on the activity of PP2B (Pandey et al., 2014). We therefore investigated whether the recycling of mGluR1 subsequent to the dopamine-mediated internalization is also dependent on the activity of protein phosphatases. Primary hippocampal neurons expressing myc–mGluR1 were pre-treated for 30 min with either 5 nM okadaic acid (OA), a specific inhibitor of PP2A, or 1 μM FK-506, a specific inhibitor for PP2B, and the drugs were present until the end of the experiment. We observed that OA did not inhibit the recycling of myc–mGluR1, whereas application of FK-506 completely inhibited the recycling of myc–mGluR1 subsequent to the dopamine-mediated internalization of the receptor (endocytosis index: control, 1.0±0.22; 30 min, 1.81±0.46; 1 h, 1.04±0.22; 1 h+OA, 1.1±0.28; 1 h+FK-506, 1.76±0.56) (Fig. 7A,B). These experiments suggest that the recycling of myc–mGluR1 following dopamine-mediated internalization is completely dependent on the activity of PP2B and does not depend on the activity of PP2A.

**Fig. 7.**
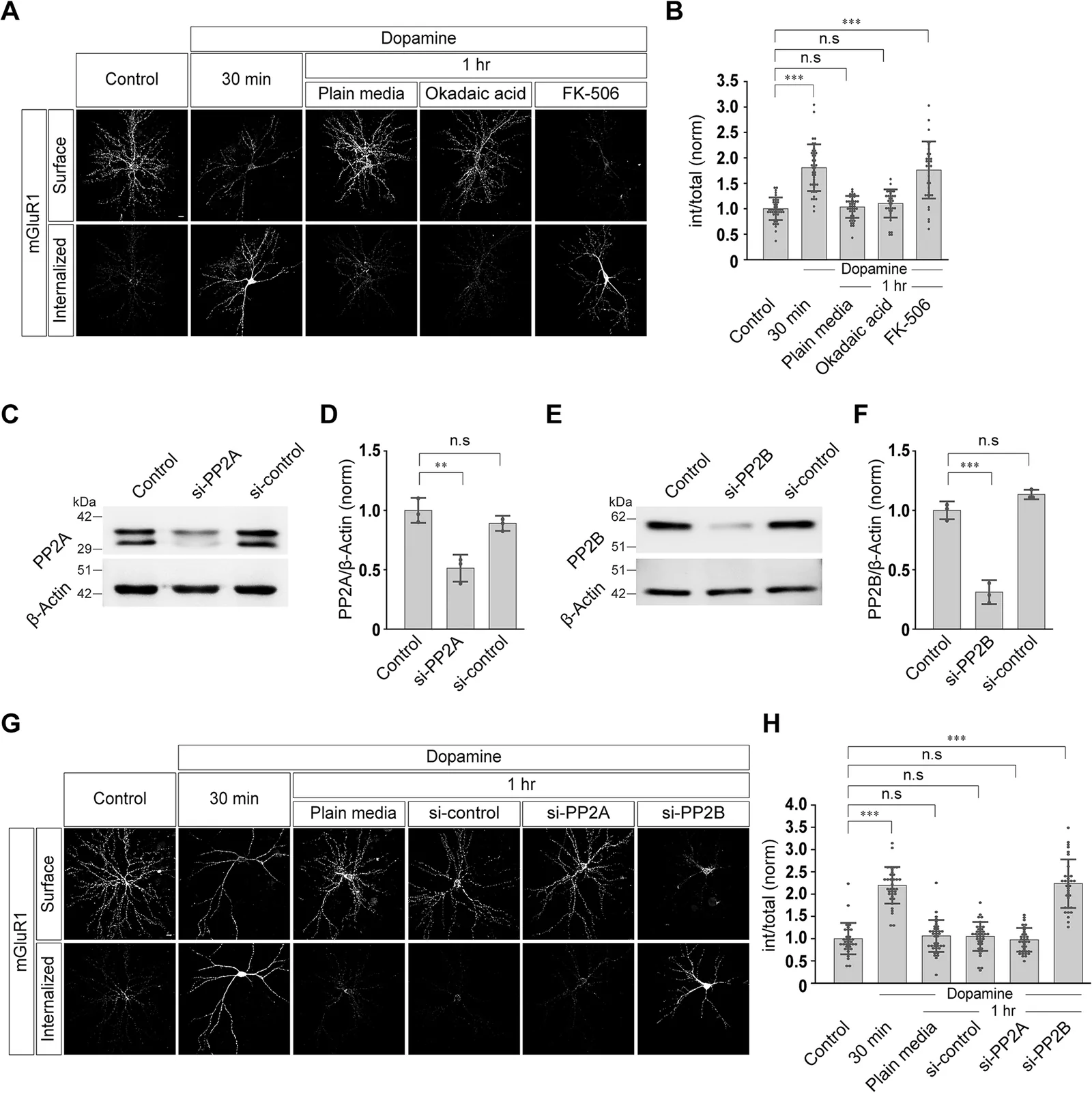
**Recycling of mGluR1 subsequent to the dopamine-mediated internalization is PP2B dependent.** (A,B) Representative images (A) and quantification of the endocytosis index (B) suggested that myc–mGluR1 recycled back to the cell surface in 1 h subsequent to the dopamine-mediated internalization. Treating cells with 5 nM okadaic acid had no effect on the recycling of the receptor but application of 1 μM FK-506 resulted in the inhibition of the recycling of myc–mGluR1 (*P*=1.362×10^−16^ between control and 30 min; *P*=0.457 between control and 1 h; *P*=0.076 between control and 1 h+OA; *P*=8.39×10^−12^ between control and 1 h+FK-506; one-way ANOVA followed by Tukey's post-test). (C,D) Western blot analyses (C) and quantification of the western blots (D) suggest efficient knockdown of the endogenous PP2A subunit PPP2CA by si-PP2A (*P*=0.006 between control and si-PP2A; *P*=0.197 between control and si-control; one-way ANOVA followed by Tukey's post-test). (E,F) Knockdown of the endogenous PP2B subunit PPP3CA by si-PP2B, as shown by the western blot (E) and quantification of the western blots (F) (*P*=6.81×10^−4^ between control and si-PP2B; *P*=0.054 between control and si-control; one-way ANOVA followed by Tukey's post-test). (G,H) Representative images (G) and quantification (H) show that acute knockdown of the endogenous PP2A subunit by si-PP2A had no effect on the recycling of the receptor. In contrast, when cells were treated with si-PP2B, it completely blocked the recycling of the receptor, whereas si-control treatment had no effect on this phenomenon (*P*=6.255×10^−20^ between control and 30 min; *P*=0.484 between control and 1 h; *P*=0.53 between control and 1 h+si-control; *P*=0.716 between control and 1 h+si-PP2A; *P*=1.921×10^−16^ between control and 1 h+si-PP2B; one-way ANOVA followed by Tukey's post-test). All results are presented as mean±s.d. collected from three independent experiments. norm, normalized. Scale bars: 10 μm. n.s, not significant, *P*>0.05; **P<0.01; ***P<0.001.

We further confirmed the effect of PP2A and PP2B on the recycling of myc–mGluR1 following dopamine-mediated internalization by acute knockdown of the endogenous PP2A and PP2B in primary hippocampal neurons. Cells were co-transfected with the myc–mGluR1 construct and siRNA against genes encoding either the endogenous PP2A subunit PPP2CA (si-PP2A) or the endogenous PP2B subunit PPP3CA (si-PP2B). Knockdown of the endogenous PP2A and PP2B subunits was studied by western blot analyses 72 h post transfection as described in the Materials and Methods. Our data suggested that si-PP2A efficiently knocked down the endogenous PP2A subunit PPP2CA (normalized protein levels: control, 1.0±0.1; si-PP2A, 0.51±0.11; si-control, 0.89±0.06) (Fig. 7C,D). Similarly, si-PP2B also efficiently knocked down the endogenous PP2B subunit PPP3CA (normalized protein levels: control, 1.0±0.07; si-PP2B, 0.31±0.1; si-control, 1.13±0.04) (Fig. 7E,F). Subsequently, we investigated the effect of knockdown of the endogenous PP2A and PP2B subunits on the recycling of dopamine-mediated internalized myc–mGluR1. Knockdown of the endogenous PP2A subunit by si-PP2A did not have any effect on the recycling of myc–mGluR1. As expected, application of 100 nM dopamine resulted in the internalization of myc–mGluR1 and the receptors subsequently recycled back to the cell surface in 1 h in control cells. myc–mGluR1 also recycled back to the cell surface in 1 h following dopamine-mediated internalization in both scrambled siRNA-transfected cells (si-control) and si-PP2A-transfected cells (endocytosis index: control, 1.0±0.35; 30 min, 2.2±0.41; 1 h, 1.06±0.36; 1 h+si-control, 1.05±0.32; 1 h+si-PP2A, 0.97±0.26) (Fig. 7G,H). Importantly, knockdown of the endogenous PP2B subunit by si-PP2B led to the complete inhibition of the recycling of myc–mGluR1 following dopamine-mediated internalization (1 h+si-PP2B, 2.23±0.54) (Fig. 7G,H). Thus, all the above experiments suggest that recycling of myc–mGluR1 subsequent to the dopamine-mediated internalization is PP2B-dependent and it does not depend on the activity of PP2A.

### Activation of group I mGluRs by dopamine triggers endocytosis of synaptic AMPARs

Rapid endocytosis of surface AMPARs can be triggered in cultured hippocampal neurons by application of glutamate receptor agonists, including glutamate itself, NMDA, AMPA and group I mGluR agonists. mGluR-mediated AMPAR endocytosis is believed to be the cellular correlate for the mGluR-dependent synaptic plasticity (Bhattacharyya, 2016; Citri and Malenka, 2008; de León-López et al., 2025; Wu et al., 2022). Because the goal of this study was to elucidate the role of dopamine in the trafficking of group I mGluRs and mGluR-triggered endocytosis of AMPARs, we studied the role of dopamine in mGluR-dependent AMPAR endocytosis. Consistent with previous results, brief application of DHPG (100 μM for 5 min) in the presence of 1 μM tetrodotoxin citrate (TTX; pre-synaptic release blocker), 20 μM 6,7-dinitroquinoxaline-2,3-dione (DNQX; AMPAR antagonist) and 50 μM D-(−)-2-amino-5-phosphonopentanoic acid (APV; NMDA receptor antagonist) caused substantial internalization of GluA1 (GRIA1)-containing receptors in primary hippocampal neurons (endocytosis index: control, 1.0±0.32; DHPG, 1.76±0.46) (Fig. 8A,B). Importantly, application of 100 nM dopamine for 5 min also caused substantial internalization of GluA1-containing receptors (dopamine, 1.84±0.43) (Fig. 8A,B). Dopamine-induced endocytosis of GluA1-containing receptors was not affected when this experiment was performed in presence of the dopamine receptor blockers. Application of 100 nM dopamine for 5 min caused internalization of GluA1-containing receptors in the presence of dopamine receptor blockers (10 μM chlorpromazine hydrochloride and 10 μM haloperidol) as well, suggesting that dopamine activated group I mGluRs, which in turn induced AMPAR endocytosis (endocytosis index: control, 1.0±0.28; dopamine, 1.85±0.28; dopamine+blockers, 1.96±0.37) (Fig. 8C,D). Application of dopamine receptor blockers alone did not cause internalization of GluA1-containing receptors (Fig. S5A,B). As dopamine-mediated internalization of mGluR1 and mGluR5 was inhibited by their respective NAMs, we investigated whether the dopamine-induced group I mGluR-mediated AMPAR endocytosis was also blocked by the group I mGluR non-competitive antagonists. Our results suggested that the dopamine-induced group I mGluR-mediated AMPAR endocytosis was inhibited upon application of 30 μM JNJ16259685 and 100 μM MTEP (endocytosis index: control, 1.0±0.31; dopamine, 1.74±0.33; dopamine+NAMs, 1.07±0.17) (Fig. 8E,F).

**Fig. 8.**
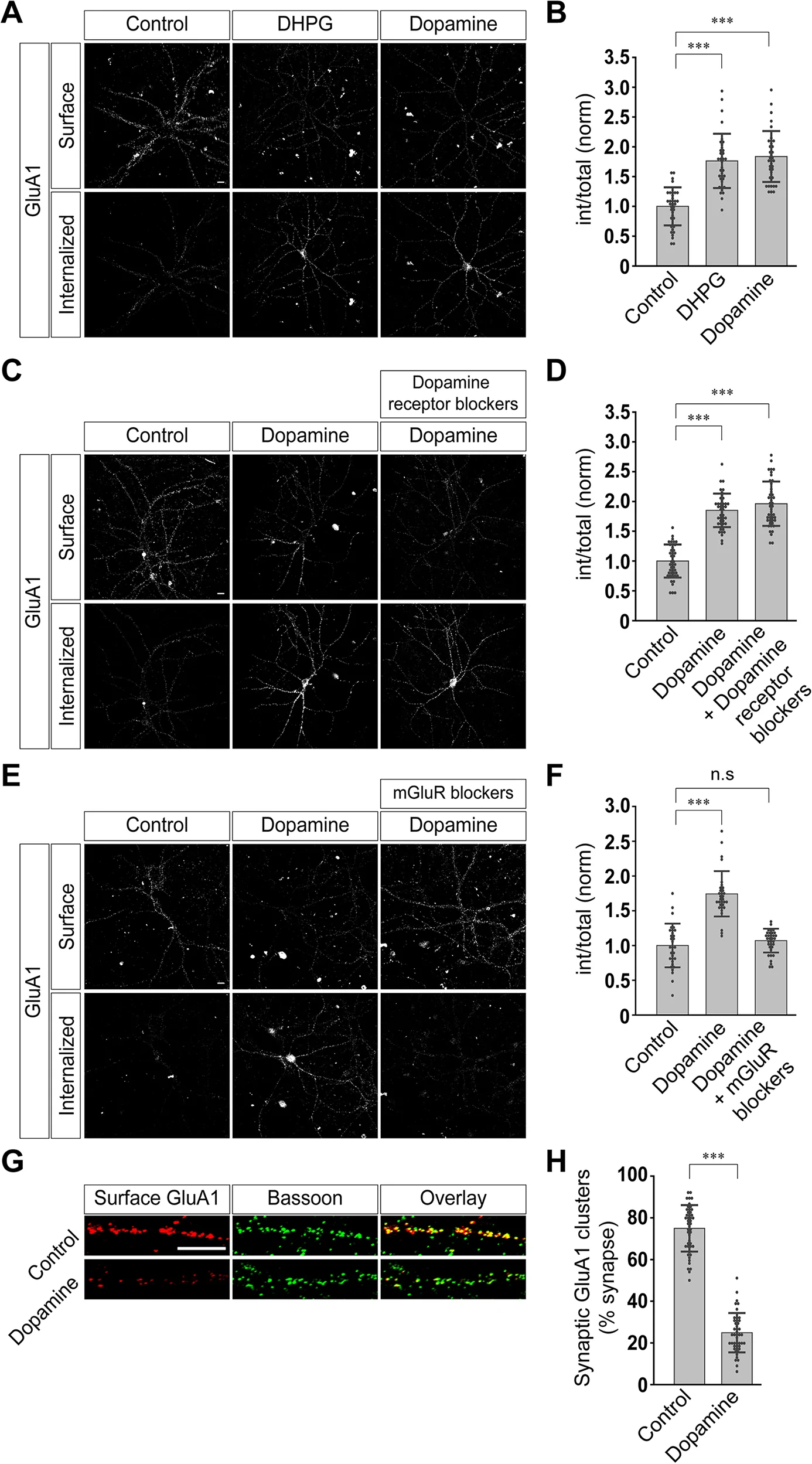
**Dopamine induces group I mGluR-mediated internalization of synaptic AMPA receptors.** (A,B) Representative images (A) and quantification of the endocytosis index (B) showing the endocytosis of GluA1-containing receptors following application of both 100 μM DHPG and 100 nM dopamine (*P*=7.052×10^−12^ between control and DHPG; *P*=4.825×10^−14^ between control and dopamine; one-way ANOVA followed by Tukey's post-test). (C,D) Application of 100 nM dopamine leads to the mGluR-mediated endocytosis of GluA1-containing receptors in presence of the dopamine receptor blockers, as shown in the representative images (C) and quantification of the internalization (D) (*P*=3.527×10^−24^ between control and dopamine; *P*=2.475×10^−23^ between control and dopamine+blockers; one-way ANOVA followed by Tukey's post-test). (E,F) Representative images (E) and quantification of the internalization index (F) suggested that non-competitive antagonists of mGluR1 and mGluR5 inhibited the 100 nM dopamine induced mGluR-mediated endocytosis of GluA1-containing receptors (F) (*P*=3.967×10^−13^ between control and dopamine; *P*=0.261 between control and dopamine+NAMs; one-way ANOVA followed by Tukey's post-test). (G) Examples of dendritic staining for surface GluA1 and bassoon after dopamine treatment. (H) Quantification of the percentage of synapses (defined by bassoon staining) that expressed detectable surface GluA1 puncta in control cells and cells that received dopamine treatment (*P*=3.307×10^−40^ between control and dopamine; unpaired *t*-test). All results are presented as mean±s.d. collected from three independent experiments. norm, normalized. Scale bars: 10 μm. n.s, not significant, *P*>0.05; ***P<0.001.

Because it was important to establish whether group I mGluR activation by dopamine led to the loss of synaptic AMPARs under our experimental conditions, we quantified the proportion of synapses containing detectable levels of surface GluA1-containing receptors by staining for surface GluA1 clusters and counterstaining for bassoon (BSN), a core component of the active zone that is commonly used to identify presynaptic terminals (Regalado et al., 2006). Brief application of dopamine (100 nM for 5 min) in the presence of all the blockers mentioned above substantially reduced the proportion of bassoon puncta that colocalized with surface GluA1 puncta (percentage of synapses with surface GluA1 puncta: control, 74.93±11.13%; dopamine, 24.91±9.43%) (Fig. 8G,H). These results suggest that dopamine induces group I mGluR-mediated internalization of synaptic AMPARs.

## DISCUSSION

Group I mGluRs not only play important roles in synaptic function and plasticity, in the last few years, they have also emerged as potential targets for the treatment of a number of neuropsychiatric disorders, including fragile X syndrome, autism and schizophrenia (Bear et al., 2004; Bhattacharyya, 2016; Citri and Malenka, 2008; Dölen et al., 2007). Trafficking of these receptors plays a pivotal role in controlling their spatiotemporal localization in the neuron, which is critical for the normal ligand–receptor interaction. Trafficking also regulates the activity of these receptors (Bhattacharyya, 2016; Dhami and Ferguson, 2006; Mahato et al., 2018). The hippocampus receives dopamine projections from the ventral tegmental area and the *substantia nigra pars compacta* (Bischoff et al., 1979a,b; Gasbarri et al., 1994a,b; Verney et al., 1985). This region also contains glutamate as the major excitatory neurotransmitter and its receptors are predominantly present in the hippocampal neurons. Dopamine has been reported to play a critical role in learning and memory formation in the hippocampus, and specific lesion of dopaminergic neurons in this region inhibits spatial learning in rats (Gasbarri et al., 1996; Lisman and Otmakhova, 2001). Interestingly, coactivation of midbrain dopamine areas and the hippocampus memory formation has been observed in fMRI studies on humans (Adcock et al., 2006; Wittmann et al., 2005). All the above evidence suggests that dopamine regulates group I mGluRs in the hippocampus. Although some amount of work has been done on agonist-mediated trafficking of group I mGluRs, the dopamine-mediated trafficking of these receptors, if any, and its physiological significance has not been investigated. In this study, we reported a novel role for dopamine in the internalization of group I mGluRs and mGluR-dependent synaptic AMPAR endocytosis, which is the cellular correlate for mGluR-mediated synaptic plasticity.

We found that dopamine induced internalization of both members of the group I mGluR family, mGluR1 and mGluR5, in heterologous HEK293 cells. HEK293 cells are one of the most widely used cell lines for heterologous expression and study of GPCRs in isolation. Furthermore, these cells do not express mGluRs or dopamine receptors (Atwood et al., 2011; Thomas and Smart, 2005). Our results in HEK293 cells suggest that dopamine induces internalization of group I mGluRs, probably through its direct interaction with the receptor. However, in order to know whether dopamine binds directly to group I mGluRs and activates them directly, more experiments need to be performed in future. Similarly, application of dopamine led to the internalization of both mGluR1 and mGluR5 in primary hippocampal neurons even in the presence of the dopamine receptor blockers, suggesting that dopamine-mediated internalization of group I mGluRs was not due to the activation of dopamine receptors by dopamine. Furthermore, the non-competitive antagonists of mGluR1 and mGluR5 inhibited the dopamine-mediated internalization of the respective receptors. Both the non-competitive antagonists of the receptors used in this study (JNJ16259685 for mGluR1 and MTEP for mGluR5) bind to an allosteric site within the seven transmembrane regions of the receptors, distinct from the orthosteric glutamate-binding region of the receptors. Binding of these non-competitive antagonists to the receptor might result in a conformational change in the receptor so that either the agonist can no longer bind effectively with the receptor or it cannot fully activate the receptor. This hypothesis needs to be experimentally investigated in the future. It has been reported that the agonist-mediated internalization of mGluR1 is arrestin and dynamin dependent (Mundell et al., 2001). Our data suggested that dopamine-mediated internalization of mGluR1 is clathrin dependent but dynamin independent. Although the clathrin-dependent and dynamin-independent endocytic pathway is a non-canonical pathway, recent studies have shed light on various mechanisms for dynamin-independent endocytosis of multiple receptors (Ferguson and Subramanian, 2018; Gundu et al., 2022; Thottacherry et al., 2018). These studies have demonstrated the biological significance of dynamin-independent endocytosis and have attracted considerable research interest. The physiological implications of dynamin-independent internalization of group I mGluRs upon application of dopamine in the hippocampus would be an important area of study in the future. Interestingly, group I mGluRs increase Gα_q_ activity and phosphorylation of ERK1/2 subsequent to the application of dopamine, similar to what was observed when the agonist DHPG was applied. The non-competitive antagonists of group I mGluRs and inhibition of Gα_q_ activity leads to the inhibition of the increase in the phosphorylation of ERK1/2 upon application of dopamine.

Following dopamine-mediated endocytosis, the majority of the internalized mGluR1 was observed to be located at the Rab11-positive recycling compartment and no significant increase in the lysosomal localization of the receptor was noticed. Interestingly, mGluR1 recycled back to the cell surface 1 h post dopamine-mediated internalization. Similarly, the other member of the group I mGluR family, mGluR5, also recycled to the cell surface in 1 h following dopamine-mediated internalization. We have reported earlier that mGluR1 recycles back to the cell surface at 2.5 h subsequent to agonist-mediated internalization, and mGluR5 takes around 3 h (Mahato et al., 2015; Pandey et al., 2014). Thus, these results suggest that group I mGluRs undergo faster recycling following dopamine-mediated internalization compared to when they are internalized by the agonist. The cause for faster recycling of these receptors subsequent to dopamine-mediated internalization needs to be investigated in future. Based on a few studies, a model has emerged that predicts that endocytosis of some GPCRs is essential for their resensitization as these GPCRs must recycle through the recycling compartments for dephosphorylation and resensitization (Bhattacharyya, 2016; Ferguson, 2001; Mahato et al., 2018). We have reported earlier that PP2A plays a critical role in the recycling of mGluR1 subsequent to its internalization by the agonist (Pandey et al., 2014). We therefore studied the role of endosomal phosphatases in the recycling of dopamine-mediated internalized mGluR1. Our data suggest that activity of PP2B is critical for the recycling of mGluR1 subsequent to its internalization by dopamine. Thus, these results indicate that, although some aspects of the internalization and recycling might be similar, there are also fundamental differences in the mechanisms of agonist- and dopamine-mediated internalization and recycling of group I mGluRs.

The detailed molecular mechanisms underlying the trafficking of AMPARs into and out of synapses are of great interest because of their importance in prominent forms of synaptic and experience-dependent plasticity (Citri and Malenka, 2008; Malenka and Bear, 2004; Shepherd and Huganir, 2007). We therefore investigated whether dopamine could induce mGluR-mediated synaptic AMPAR endocytosis, the cellular correlate for mGluR-dependent synaptic plasticity in hippocampal neurons. Our data presented here suggest that dopamine plays a critical role in the regulation of mGluR-dependent AMPAR endocytosis. Furthermore, we report here that application of dopamine leads to mGluR-mediated AMPAR endocytosis even in the presence of dopamine receptors blockers, suggesting that dopamine does not induce mGluR-mediated AMPAR internalization through activation by its own receptors. The majority of surface AMPAR puncta in the dendrites of cultured neurons are synaptic, as defined by colocalization with presynaptic or postsynaptic markers. It thus seems likely that the internalized AMPARs in the dendrites were predominantly of synaptic origin. Indeed, our results confirmed that brief application of dopamine caused the mGluR-mediated internalization of synaptic AMPARs. In summary, the regulation of group I mGluR trafficking and mGluR-mediated AMPAR endocytosis by dopamine adds a completely new dimension in the interaction of dopaminergic and glutamatergic systems in the hippocampus. Such interactions could have enormous implications in understanding normal brain function as well as developing a broad range of neuropsychiatric therapeutics involving dopamine and glutamate receptors.

## MATERIALS AND METHODS

### Materials

The myc–mGluR1 and myc–mGluR5 constructs were generously gifted by Katherine Roche (National Institutes of Health, USA). In these constructs, the myc epitope was tagged at the N-terminus of full-length mGluR1 and mGluR5. The FLAG–mGluR1 construct was generously provided by Johanna Montgomery (University of Auckland, New Zealand). HEK293 cells were purchased from the National Centre for Cell Science (Pune, India). DHPG, DNQX, APV, YM254890, MTEP and JNJ16259685 were purchased from Tocris (UK). TTX was purchased from Abcam (UK). Dopamine hydrochloride, chlorpromazine hydrochloride, haloperidol, pitstop 2 and dynasore hydrate were purchased from Sigma-Aldrich (USA). Dulbecco's modified Eagle's medium (DMEM), neurobasal medium, fetal bovine serum (FBS), B27, Lipofectamine 2000, antibiotic-antimycotic mix and other supplements for cell culture were purchased from Invitrogen (USA). All salts and fine chemicals such as poly-D-lysine, 5-fluoro-2′-deoxyuridine (FUDR), paraformaldehyde (PFA), polyethylenimine (PEI) and Fluoromount aqueous mounting medium were purchased from Sigma-Aldrich (USA). Protein A/G PLUS agarose beads were purchased from Santa Cruz Biotechnology (USA) and Immobilon forte western HRP substrate was purchased from Millipore (USA). ON-TARGET plus siRNA SMARTpools against clathrin heavy chain, dynamin-1, the PP2A subunit PPP2CA and the PP2B subunit PPP3CA were purchased from Horizon discovery Dharmacon (UK). The sequences of all the si-RNAs have been provided in Table S1. Anti-myc mouse monoclonal (ab18185) and anti-LAMP1 rabbit polyclonal (ab62562) antibodies were purchased from Abcam (UK). Anti-FLAG rabbit polyclonal antibody (F7425) was from Sigma-Aldrich (USA). Anti-GluA1 rabbit polyclonal antibody (ABN241) was purchased from Millipore (USA) and anti-bassoon mouse monoclonal antibody (SAP7F407) was from Enzo Life Sciences (USA). Anti-ERK1/2 mouse monoclonal (9107S), anti-phospho-ERK1/2 rabbit monoclonal (4370S) and anti-PP2A C subunit rabbit polyclonal antibodies (2038T) were purchased from Cell Signaling Technology (USA). Anti-dynamin rabbit polyclonal (PA1-660), anti-clathrin heavy chain mouse monoclonal (MA1-065) and anti-Rab11 rabbit polyclonal (71-5300) antibodies were purchased from Invitrogen (USA). Anti-PP2B mouse monoclonal (sc-17808), anti-β-actin mouse monoclonal (sc-81178) and anti-Gα_q_ mouse monoclonal (sc-136181) antibodies were obtained from Santa Cruz Biotechnology (USA). Alexa Fluor-conjugated secondary antibodies (goat anti-mouse Alexa Fluor 568, A11031; goat anti-mouse Alexa Fluor 647, A21236; goat anti-rabbit Alexa Fluor 568, A11036; goat anti-rabbit Alexa Fluor 647, A21245; goat anti-rabbit Alexa Fluor 488, A11034) were purchased from Invitrogen (USA) and horseradish peroxidase (HRP)-conjugated secondary antibodies (anti-mouse IgG-peroxidase, A9044; anti-rabbit IgG-peroxidase, A0545) were purchased from Sigma-Aldrich (USA).

### Construct preparation

The shRNA against clathrin heavy chain (sh-clathrin) and scrambled control (sh-control) were cloned individually in a multi-promoter vector (LRV1 vector, generated in-house) under the H1 promoter. The sequence of the shRNA used for this study was 5ʹ-GTTGGTGACCGTTGTTATG-3ʹ and this shRNA has been demonstrated to be effective in knocking down the endogenous clathrin in primary hippocampal neurons in a previous report (Watanabe et al., 2014). Enhanced green fluorescent protein (eGFP) expression, which was under an internal ribosome entry site (IRES) element, was used to identify transfected cells.

### HEK293 cell culture and transfection

HEK293 cells were cultured in DMEM supplemented with 10% FBS and antibiotic-antimycotic mix at 37°C with 95% humidity and 5% CO_2_. Cells were transfected on 12 mm coverslips coated with 50 μg/ml poly-D-lysine by mixing a total of 1 μg of myc–mGluR1 and myc–mGluR5 DNA with 3 μg of PEI. For co-immunoprecipitation experiments, transfections were performed in 60 mm dishes by mixing 5 μg of FLAG–mGluR1 DNA with 15 μg of PEI. PEI is a stable cationic polymer that condenses DNA into positively charged particles and the DNA–PEI complex enters into the cell through endocytosis (Sonawane et al., 2003). Experiments were carried out 24 h post transfection.

### Dissociated primary hippocampal neuron culture and transfection

Primary hippocampal neuron cultures were prepared from P0 C57BL/6 mice of either sex. Briefly, hippocampi were dissected out from the pups. The tissues were dissociated in the enzymatic solution containing papain for 30 min at 37°C. Subsequently, trituration was performed to obtain single cells. Neurons were then plated on coverslips or dishes pre-coated with poly-D-lysine in sodium borate (50 μg/ml poly-D-lysine+0.1 M sodium borate) at a density of approximately 150,000 cells per 12 mm well in a 24-well plate. Cultures were maintained in neurobasal medium supplemented with B27 and 0.5 mM glutamine. Glial growth was inhibited by adding FUDR on the fourth day of culture. All the studies were approved by the Institutional Animal Ethics Committee (IAEC) of the Indian Institute of Science Education and Research Mohali.

Primary hippocampal neurons were transfected with myc–mGluR1 or myc–mGluR5, and sh-clathrin or sh-control constructs at 7 or 8 days *in vitro* using the calcium phosphate method. For knockdown of endogenous clathrin, dynamin and PP2A and PP2B subunits with siRNA, ON-TARGET plus SMARTpool siRNA and scrambled siRNA were co-transfected with the myc–mGluR1 construct in primary hippocampal neurons at 7 or 8 days *in vitro* using Lipofectamine 2000 following the manufacturer's instructions. All experiments were performed when the cells were at 12–15 days *in vitro*.

### Endocytosis and recycling assays

For DHPG- and dopamine-mediated mGluR internalization assays, HEK293 cells or primary hippocampal neurons were transfected with either the myc–mGluR1 or myc–mGluR5 construct. Subsequently, the entire endocytosis experiments were performed in HEK293 cells or primary hippocampal neurons in plain DMEM or plain neurobasal medium (glutamate free and without B27). Live cells were incubated with anti-myc mouse monoclonal primary antibody (1:500 for HEK293 cells and 1:200 for primary hippocampal neurons) for 15 min at 37°C. Cells were then treated with either 100 μM DHPG or 100 nM dopamine for indicated times or for 5 min to induce endocytosis of the receptors. Cells were then incubated at 37°C for various time periods in plain DMEM or plain neurobasal medium in the absence of the ligand. Subsequently, cells were fixed without permeabilization using ice-cold 4% PFA for 15 min on ice. Surface-localized receptors were then labelled with a saturating concentration of goat anti-mouse Alexa Fluor 568-conjugated secondary antibody (1:100) for 1 h at 37°C. Cells were then permeabilized with 0.1% Triton X-100 for 30 min at room temperature. The endocytosed receptors were then labelled by the application of goat anti-mouse Alexa Fluor 647-conjugated secondary antibody (1:750) for 1 h at 37°C. The coverslips were mounted on glass slides and imaged under the confocal microscope. To ensure that the Alexa Fluor 647-conjugated secondary antibody did not label any detectable surface receptors in our assays, we performed control experiments to determine the saturating concentration of the first secondary antibody as described in our earlier studies (Gulia et al., 2017; Mahato et al., 2015; Pandey et al., 2014; Trivedi and Bhattacharyya, 2012). The control experiments suggested that in our assays, the Alexa Fluor 647-conjugated secondary antibody did not label any detectable amount of surface receptors and thus it stained the internalized receptors only. The role of dopamine receptor blockers, non-competitive antagonists (NAMs) of group I mGluRs and the Gα_q_ inhibitor in 100 nM dopamine-mediated internalization of myc–mGluR1 or myc–mGluR5 was studied by pre-incubating primary hippocampal neurons with the respective blockers, followed by performing the endocytosis assays using the protocol described above. The effect of knockdown of endogenous clathrin and dynamin on the 100 nM dopamine-mediated internalization of myc–mGluR1 and the effect of knockdown of the endogenous dynamin on the 100 μM DHPG-mediated internalization of myc–mGluR1 were studied by co-transfection of the primary hippocampal neurons with the myc–mGluR1 construct and siRNA against clathrin or dynamin or sh-clathrin. The endocytosis experiments were performed 72 h post transfection using the method described before.

The recycling experiments for group I mGluRs in primary hippocampal neurons were also performed in plain neurobasal medium. The recycling of myc–mGluR1 and myc–mGluR5 following dopamine-mediated internalization or DHPG-mediated internalization was studied by initially labelling the myc–mGluR1- or myc–mGluR5-expressing live primary hippocampal neurons with anti-myc primary antibody and subsequently inducing internalization of the receptor by the application of 100 nM dopamine for 5 min. Following this, cells were incubated in plain neurobasal medium for different periods in the absence of dopamine. After specific time points, cells were fixed and surface and internalized receptors were labelled by goat anti-mouse Alexa Fluor 568- and goat anti-mouse Alexa Fluor 647-conjugated secondary antibodies, respectively, using the same protocol as described for the endocytosis assay. In case of the experiments where the effect of OA and FK-506 (both Tocris, UK) in the recycling of the receptors were tested, drugs were applied 30 min before the application of the primary antibody and the drugs were present throughout the experiment. The effect of knockdown of the endogenous PP2A and PP2B subunits on the recycling of myc–mGluR1 was studied by co-transfection of primary hippocampal neurons with the myc–mGluR1 construct and siRNA against the PP2A or PP2B subunits. The recycling experiments were performed 72 h post transfection using the method described before.

Similar to the endocytosis and recycling experiments of group I mGluRs, the mGluR-mediated AMPAR endocytosis experiments in primary hippocampal neurons were also performed in plain neurobasal medium. To study the role of dopamine in the mGluR-mediated AMPAR endocytosis, cells were first pre-incubated in 1 μM TTX, 20 μM DNQX and 50 μM APV for 30 min at 37°C. For investigating the role of dopamine in the mGluR-mediated AMPAR endocytosis in the presence of dopamine receptor blockers, cells were incubated with 10 μM chlorpromazine hydrochloride and 10 μM haloperidol along with the other abovementioned inhibitors for 30 min at 37°C. Subsequently, GluA1-containing receptors were labelled in live neurons by 15 min incubation at 37°C with a rabbit polyclonal antibody directed against the N-terminus of the GluA1 subunit (1:150). Cells were then washed with plain medium and 100 nM dopamine was applied for 5 min. Dopamine was then washed out and cells were further incubated in the presence of the blockers for a total of 15 min at 37°C. Following this, cells were fixed without permeabilization using ice-cold 4% PFA for 15 min on ice. Surface receptors were then labelled by the application of saturating amount of goat anti-rabbit Alexa Fluor 568-conjugated secondary antibody (1:100) for 1 h at 37°C. Cells were then permeabilized with 0.1% Triton X-100 for 30 min at room temperature and the endocytosed receptors were labelled by the application of goat anti-rabbit Alexa Fluor 647-conjugated secondary antibody (1:750) for 1 h at 37°C. Coverslips were mounted on glass slides and scanned under the confocal microscope. The saturating concentration of the Alexa Fluor 568-conjugated secondary antibody that was used to label the surface GluA1-containing receptors was determined by performing control experiments as described in our earlier studies (Gulia et al., 2017; Pandey et al., 2020; Sharma et al., 2018). These experiments also suggested that the Alexa Fluor 647-conjugated secondary antibody that was used to label the internalized receptors did not label any detectable surface receptors. The role of non-competitive antagonists of group I mGluRs in 100 nM dopamine-induced group I mGluR-mediated AMPAR endocytosis was investigated by pre-incubating primary hippocampal neurons with antagonists (30 μM JNJ16259685 and 100 μM MTEP), followed by performing the endocytosis assays using the protocol described above.

### Colocalization assay

In order to study the fate of myc–mGluR1 subsequent to dopamine-mediated internalization, receptors were labelled in transfected primary hippocampal neurons with mouse monoclonal anti-myc primary antibody (1:200) for 15 min at 37°C. Following 5 min application of 100 nM dopamine, cells were chased for 25 min in plain medium at 37°C. Subsequently, antibodies attached to the remaining surface receptors that did not internalize on dopamine application were stripped by treating cells with ice-cold acidic solution (0.5 M NaCl, 0.2 N CH_3_COOH) for 2 min on ice. Cells were then fixed with ice-cold 4% PFA and permeabilized in 0.1% Triton X-100 for 30 min at room temperature, followed by incubation with rabbit polyclonal antibody against Rab11 (1:200) or rabbit polyclonal antibody against LAMP1 (1:500) overnight at 4°C. After that, appropriate secondary antibodies – goat anti-mouse Alexa Fluor 568 (1:750) and goat anti-rabbit Alexa Fluor 488 (1:750) – were added for 1 h at 37°C to stain the internalized myc–mGluR1 and Rab11 or LAMP1, respectively.

To investigate whether application of dopamine leads to the mGluR-mediated endocytosis of synaptic AMPARs, synaptic GluA1 puncta were quantified by colocalization of surface GluA1 puncta and presynaptic bassoon puncta, before and after the application of dopamine. Cells were first pre-incubated in 1 μM TTX, 20 μM DNQX, 50 μM APV and dopamine receptor blockers (10 μM chlorpromazine hydrochloride and 10 μM haloperidol) for 30 min at 37°C. After live-cell staining with the anti-GluA1 N-terminus rabbit polyclonal antibody, cells were either treated without dopamine (control) or 100 nM dopamine was applied as described above. Cells were then fixed in 4% PFA on ice for 15 min without permeabilization and were incubated with goat anti-rabbit Alexa Fluor 568-conjugated secondary antibody (1:100) for 1 h at 37°C. Subsequently, cells were permeabilized in 0.1% Triton X-100 for 30 min at room temperature and incubated with a monoclonal antibody against bassoon (1:500) overnight at 4°C. After this, cells were incubated with goat anti-mouse Alexa Fluor 488-conjugated secondary antibody (1:750) for bassoon puncta visualization. Coverslips were mounted on glass slides and scanned under the confocal microscope.

### Western blotting and co-immunoprecipitation assays

The knockdown efficiency of endogenous clathrin, dynamin and the PP2A and PP2B subunits by their respective siRNAs were studied by western blot analysis. For these assays, primary neurons were transfected with si-clathrin, si-dynamin, si-PP2A or si-PP2B. 72 h post-transfection, cells were washed with ice-cold 1× PBS and lysed in RIPA lysis buffer with protease inhibitor cocktail. Samples were subsequently boiled in 6× Laemmli sample buffer at 99°C for 10 min and run on SDS-polyacrylamide gels by loading equal amount of protein in each lane. Proteins were then transferred onto PVDF membranes, which were blocked with 5% BSA in PBS containing 0.05% Tween-20 for 1 h at room temperature. Membranes were then incubated with anti-clathrin (1:200), anti-dynamin (1:500), anti-PP2A (1:200), anti-PP2B (1:200) and anti-β-actin antibodies (1:1000) at 4°C overnight. β-actin was used as the loading control. After washing, membranes were incubated with the horseradish peroxidase-conjugated secondary antibodies (1:5000) for 45 min at room temperature. Blots were developed using Immobilon forte western HRP substrate and images were acquired in ImageQuant LAS 4000.

In order to investigate the ability of group I mGluRs to upregulate the phosphorylation of ERK1/2 upon application of DHPG and dopamine, primary neurons were pre-incubated with 100 μg/ml cycloheximide (Sigma) for 5 h to inhibit the synthesis of new receptors. Subsequently, 100 μM DHPG or 100 nM dopamine (in the absence and presence of the dopamine receptor blockers) was applied for 5 min, followed by fixation of cells. Cells were then lysed and samples were run on SDS-polyacrylamide gels, followed by western blotting using the same protocol as described above. Phospho-ERK1/2 and total ERK1/2 immunoblotting was performed using anti-phospho-ERK1/2 antibody (1:1000) and anti-ERK1/2 antibody (1:1000), respectively. The effect of non-competitive antagonists of group I mGluRs and Gα_q_ inhibitor on the group I mGluR-mediated upregulation of the phosphorylation of ERK1/2 upon application of 100 nM dopamine in HEK293 cells and primary neurons was investigated by first pre-incubating the myc–mGluR1-expressing HEK293 cells or primary neurons with 100 μg/ml cycloheximide for 5 h to inhibit the synthesis of new receptors, followed by treating the cells with either group I mGluR non-competitive antagonists (30 μM JNJ16259685 for mGluR1 and 100 μM MTEP for mGluR5) or Gα_q_ inhibitor (10 μM YM254890). Subsequently, 100 nM dopamine was applied for 5 min and cells were processed using the same protocol described above. Immunoblotting was performed using anti-phospho-ERK1/2 antibody (1:1000) and anti-ERK1/2 antibody (1:1000).

In order to determine whether application of dopamine leads to the increased interaction of mGluR1 with Gα_q_ protein, co-immunoprecipitation experiments were performed. HEK293 cells were transfected with FLAG–mGluR1 cDNA. 24 h post transfection, cells were treated with 100 μM DHPG or 100 nM dopamine for 2 min and then fixed with ice-cold 4% PFA for 15 min. Subsequently, cells were lysed using TAP lysis buffer (20 mM Tris, pH 8.0, 150 mM NaCl, 0.5% NP-40, 1 mM MgCl_2_, 1 mM Na_3_VO_4_ and 1× protease inhibitor cocktail). The lysate was centrifuged at 21,200 ***g*** for 30 min. 50 μl of supernatant was collected as input. Immunoprecipitation was performed by incubating the remaining supernatant with protein A/G beads that were prepared by overnight incubation with anti-FLAG primary antibody. After 6–8 h, beads were washed and samples were boiled in 2× Laemmli buffer. The samples were then run on SDS-polyacrylamide gels, followed by western blotting using the method described above. For immunoblotting, antibodies against Gα_q_ (1:200) and FLAG (1:300) were used.

### Image acquisition and analysis

For image acquisition, coverslips were mounted in Fluoromount mounting medium. Imaging was done using a Nikon A1 confocal laser scanning microscope with a 60× oil immersion objective (NA=1.4). Images from all the conditions in a particular experiment were obtained using identical parameters. All analyses were done in a masked manner using raw images, and quantification was done using ImageJ software (National Institutes of Health, USA) (Schneider et al., 2012). All the analytical procedures have been described in our previous studies (Gulia et al., 2017; Mahato et al., 2015; Ojha et al., 2022; Pandey et al., 2014, 2020; Sharma et al., 2018; Trivedi and Bhattacharyya, 2012). Briefly, raw images from each experiment were maximally projected and thresholded using equal values across all comparative images to avoid bias. Thresholding cells for quantification separates real fluorescence from the background by creating a binary mask typically based on pixel intensity histograms, to enable accurate measurement of intensity or area. Following this, the thresholded areas of fluorescently labelled surface and internalized receptors were measured. Internalization index for each cell was then calculated by dividing the value contributed by the internal fluorescence with the value contributed by the total fluorescence (surface+internal). These values were then normalized with respect to their controls. To measure the surface receptors, raw images from individual experiments were maximally projected and thresholded for surface fluorescence measurements using identical values for a particular experimental condition. The thresholded areas of fluorescently labelled surface receptors were divided by the cell area, which was determined by measuring the background fluorescence using a low threshold level. These values were then normalized to the average fluorescence of control cells. For primary neurons, all the results represent dendritic values, which were defined by the area that was 10 μm away from the soma. For colocalization experiments, the colocalization was quantified at a particular *z*-section of the image after thresholding the cells using identical values. To obtain representative images, raw images were processed using Adobe Photoshop software (Adobe Systems) by using identical values of brightness and contrast. All the western blots and immunoprecipitation experiments were also quantified using ImageJ software. Briefly, densitometric analysis was performed by selecting each band, followed by signal intensity quantification. Data were acquired as percentage area values and individual band intensities were normalized to β-actin in the case of western blots, or to the pulled-down protein in the case of co-immunoprecipitation. The extent of colocalization of internalized myc–mGluR1 with Rab11 or LAMP1 was studied along 50 μm portions of dendrites. Synaptic GluA1 puncta were defined by visual colocalization with presynaptic bassoon puncta in 50 μm portions of dendrites.

### Statistical analysis

Each experiment was repeated three times and a total of 30–40 primary hippocampal neurons were imaged in each condition. The quantification of an experiment has been represented as a combined result for all the repeats of that particular experiment. Data are presented as mean±s.d. Experimental group results were compared with each other using two-tailed unpaired Student's *t*-test or one-way ANOVA followed by Tukey's post-test. *P*>0.05 was considered as non-significant.

## Supplementary Material



10.1242/joces.264725_sup1 Supplementary information
