# Supplementary material for: Metabotropic glutamate receptor internalization and synaptic AMPA receptor endocytosis by dopamine
Source: J Cell Sci. 2026 Aug 13;139(15):jcs264725. doi: 10.1242/jcs.264725 (PMC13728498; doi:10.1242/jcs.264725)
Supplement: Supplementary file 1 [file joces-139-264725-s1.pdf]

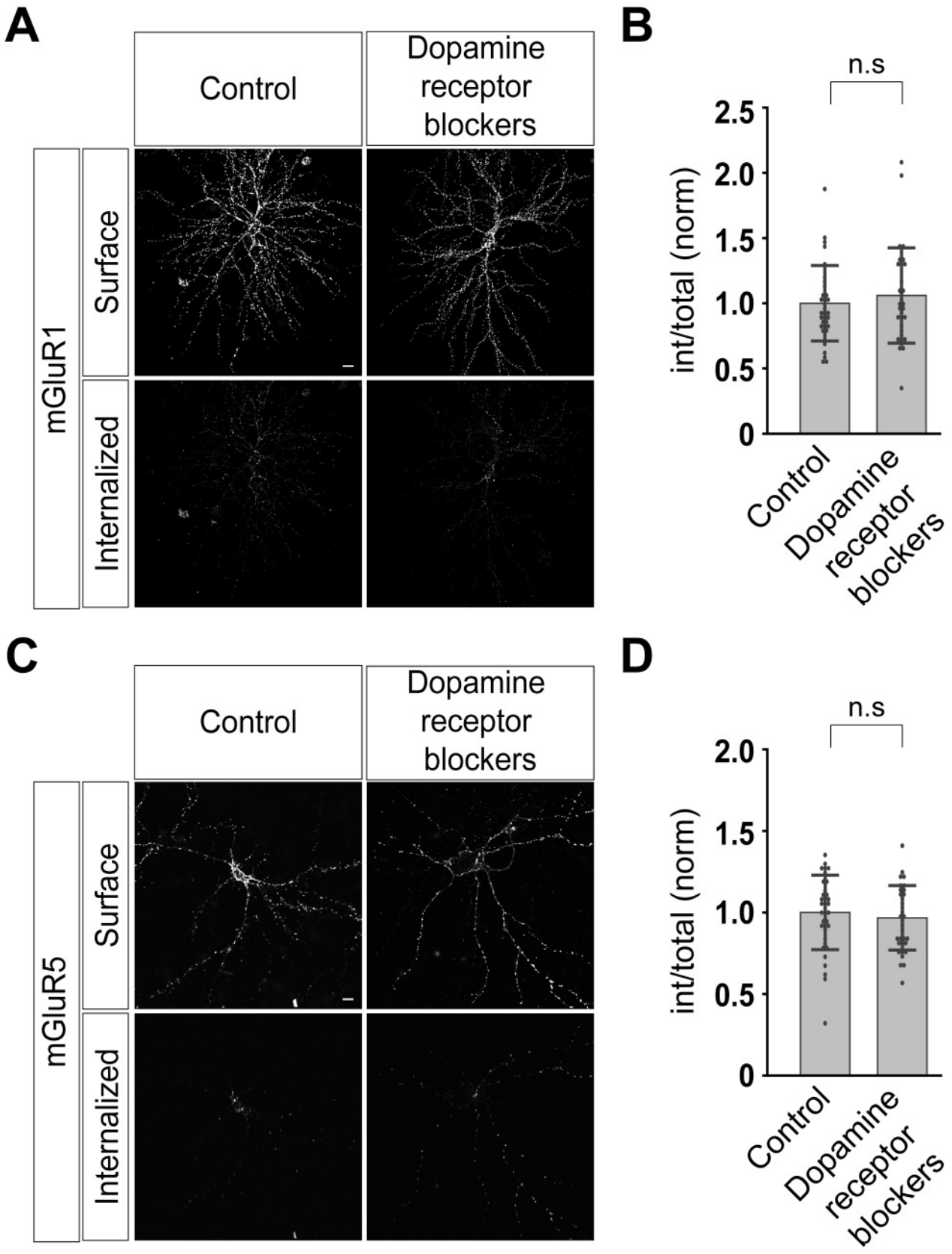

Figure S1

Fig. S1. Dopamine receptor blockers do not induce internalization of mGluR1 and mGluR5. (A, B) Representative images (A) and quantitation (B) suggest that dopamine receptor blockers alone did not cause internalization of myc-mGluR1 in primary hippocampal neurons. (C, D) Application of dopamine receptor blockers did not induce internalization of myc-mGluR5 in primary hippocampal neurons as shown in representative images (C) and quantitation (D). Results are presented as means ± SD collected from three independent experiments. Scale bar = 10 µm. n.s, $p > 0.05$.

Journal of Cell Science • Supplementary information

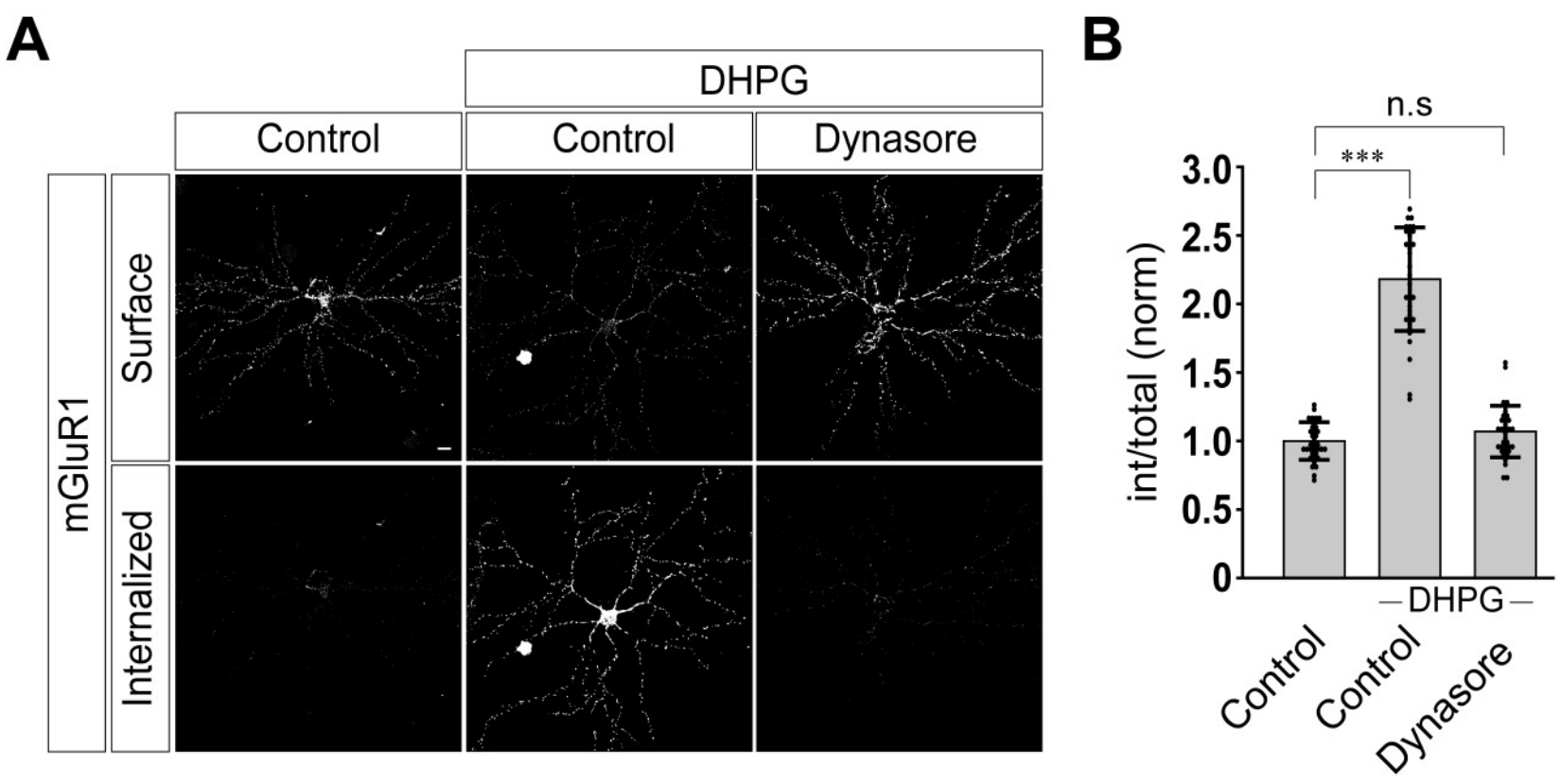

Figure S2

Fig. S2. Agonist-mediated internalization of mGluR1 is dynamin-dependent. (A, B) R,S-DHPG-mediated internalization of myc-mGluR1 was inhibited upon inhibition of dynamin by dynasore in primary hippocampal neurons as shown by the representative images (A) and quantitation of the endocytosis index (B). All the results represented as means ± SD collected from three independent experiments. Scale bar = 10 µm. ***, $p < 0.001$; n.s, $p > 0.05$.

Journal of Cell Science • Supplementary information

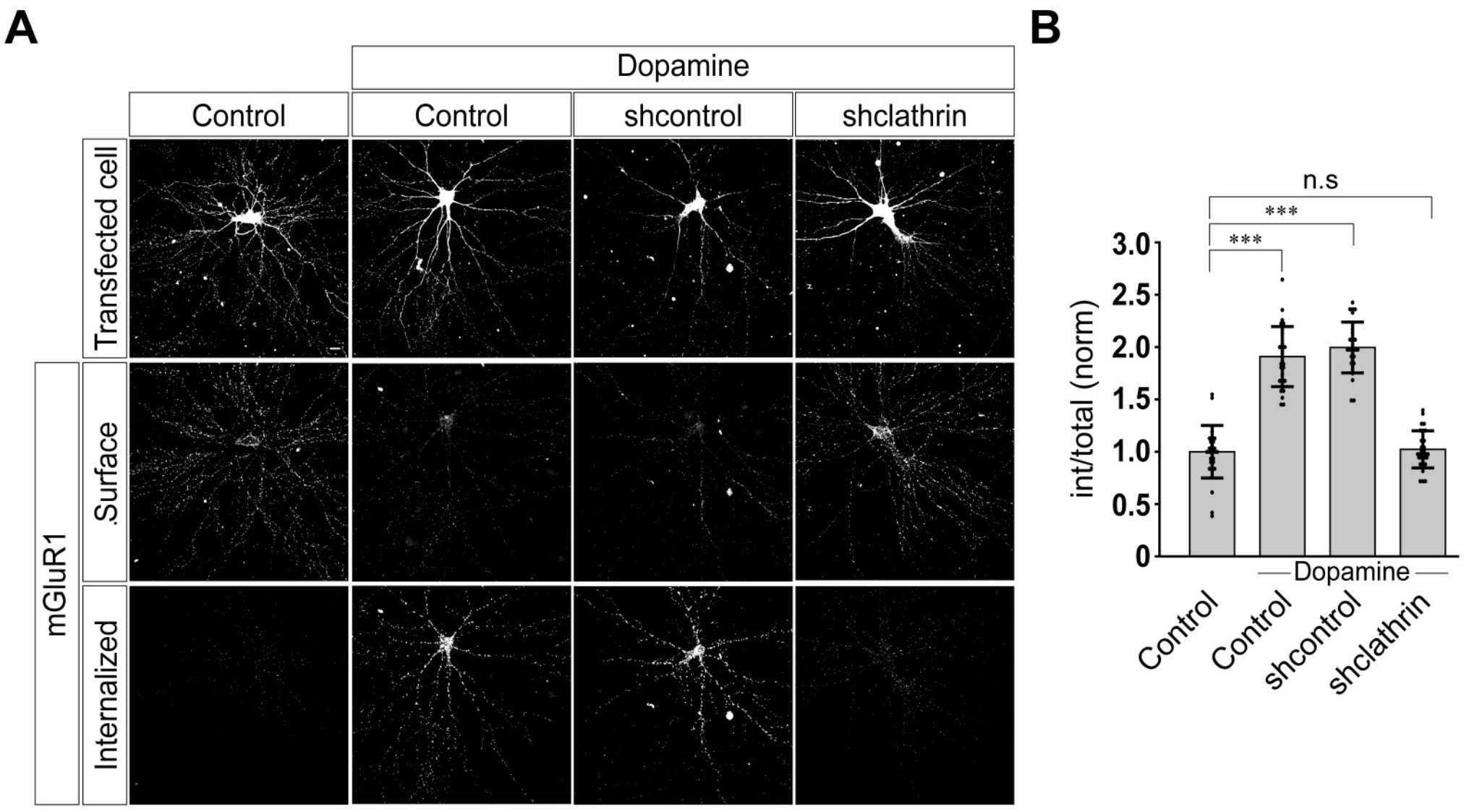

Figure S3

Fig. S3. Dopamine-mediated internalization of mGluR1 is clathrin-dependent. (A, B) Representative images (A) and quantitation of the endocytosis index (B) suggested that knockdown of the endogenous clathrin by shclathrin inhibited the 100 nM dopamine-mediated internalization of myc-mGluR1 in primary hippocampal neurons. On the other hand, in control cells and shcontrol transfected cells the receptor showed normal internalization upon dopamine application. All the results represented as means ± SD collected from three independent experiments. Scale bar = 10 μm. ***, $p < 0.001$; n.s, $p > 0.05$.

Journal of Cell Science • Supplementary information

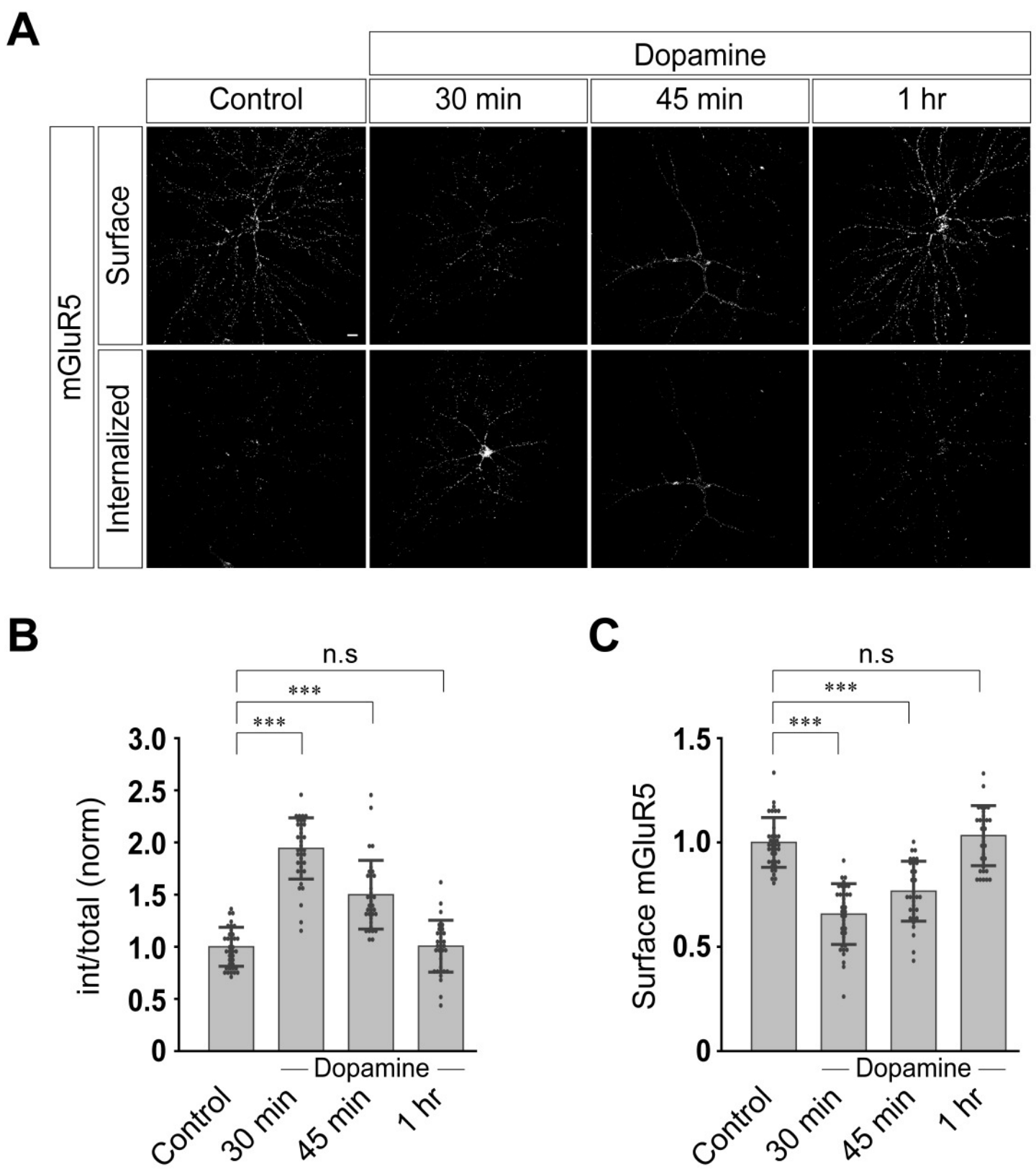

Figure S4

Fig. S4. Recycling of mGluR5 to the cell surface subsequent to the dopamine-mediated internalization. (A) Representative images showing the recycling of dopamine-induced internalized myc-mGluR5 at the cell surface in primary hippocampal neurons. (B, C) myc-mGluR5 internalized upon application of 100 nM dopamine and the receptor recycled back to the cell surface in 1 hr as shown by the quantitation of the endocytosis index (B) as well as measurement of the surface myc-mGluR5 (C). All the results represented as means ± SD collected from three independent experiments. Scale bar = 10 µm. ***, $p < 0.001$; n.s, $p > 0.05$.

Journal of Cell Science • Supplementary information

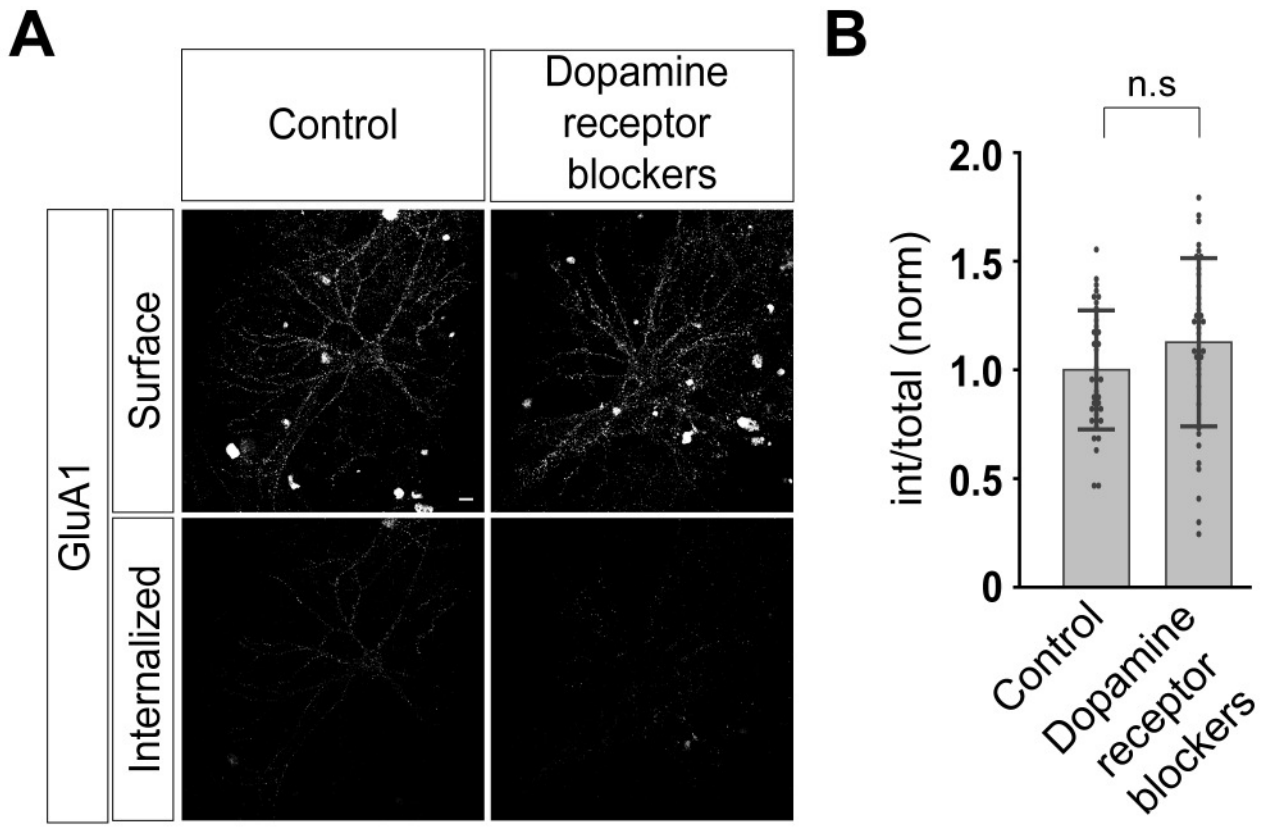

Figure S5

Fig. S5. Dopamine receptor blockers do not induce internalization of GluA1-containing receptors. (A, B) Representative images (A) and quantitation of the internalization index (B) show that application of dopamine receptor blockers alone did not induce internalization of the GluA1-containing receptors in primary hippocampal neurons. Results are presented as means ± SD collected from three independent experiments. Scale bar = 10 μm. n.s, $p > 0.05$.

Table S1. Sequences of siRNAs and shRNAs

S. No	Target	Oligo type	Sequence (5' ⟶3')
1.	PP2A	ON-TARGET plus siRNA	1. CGACGAGUGUUUAAGGAAA<br>2. CCACAUGUCACUCGUCGUA<br>3. CCAAACUAUUGCUAUCGUU<br>4. GAGGAUAUUACUCUGUUGA
2.	PP2B	ON-TARGET plus siRNA	1. GUUCGUACUUCUACAGUUA<br>2. CGUACUGGCUCCCAAAUUU<br>3. ACAGAGUAUUUCACGUUUA<br>4. UAACAGAGGGUGCUUCGAU
3.	Dynamin-1	ON-TARGET plus siRNA	1. GCGUGUACCCUGAGCGUGU<br>2. UGGUAUUGCUCCUGCGACA<br>3. GGGAGGAGAUGGAGCGAAU<br>4. GCUGAGACCGAUCGAGUCA
4.	Clathrin	ON-TARGET plus siRNA	1. GGAAAGCAAUCCAUACAGA<br>2. UCAGAAGAAUUGCUGCUUA<br>3. GACAAAUUCAUCUGCAUUA<br>4. ACAAAGCAAUCCAGUUCUA
5.	Non-targeting pool	ON-TARGET plus siRNA	1. UGGUUUACAUGUCGACUAA<br>2. UGGUUUACAUGUUGUGUGA<br>3. UGGUUUACAUGUUUUCUGA<br>4. UGGUUUACAUGUUUUCCUA
6.	Clathrin	shRNA	GTTGGTGACCGTTGTTATG
7.	Scrambled control	shRNA	TTCGCACCCTACTTCGTGG

Journal of Cell Science • Supplementary information
