# Supplementary material for: Metabotropic glutamate receptor internalization and synaptic AMPA receptor endocytosis by dopamine
Source: J Cell Sci. 2026 Aug 13;139(15):jcs264725. doi: 10.1242/jcs.264725 (PMC13728498; doi:10.1242/jcs.264725)
Supplement: Supplementary file 2 [file joces-139-264725_review_history.pdf]

Metabotropic glutamate receptor internalization and synaptic AMPA receptor endocytosis by dopamine

K. Aruna, Mitra Kulkarni and Samarjit Bhattacharyya
DOI: 10.1242/jcs.264725

Editor: Subhojit Roy

Review timeline

Original submission:	15 January 2026
Editorial decision:	2 March 2026
First revision received:	23 May 2026
Accepted:	8 July 2026

Original submission

First decision letter

MS ID#: jcs.264725

MS TITLE: Metabotropic glutamate receptor internalization and synaptic AMPA receptor endocytosis by dopamine

AUTHORS: K Aruna; Mitra Kulkarni; Samarjit Bhattacharyya
ARTICLE TYPE: Research Article

Dear Dr Bhattacharyya,

Thank you for your recent submission to Journal of Cell Science and apologies for the delay because I had a hard time recruiting reviewers. Unfortunately, reviewer 1 has a number of conceptual and technical concerns that seem legitimate. In my opinion they will be difficult to address, even with more experiments. Thus I don't see a clear path forward for this paper in JCS, and feel that submission elsewhere may be more appropriate. However, if you still feel that these concerns can be addressed, you may choose to resubmit. In that case, I would like to let you know that there must be support from the reviewers for the paper to move forward.

Reviewer 1

Review of Aruna et al. for Journal of Cell Science

In this manuscript, the authors report that dopamine directly activates and promotes internalization of mGluR1/5 in HEK293 cells and primary hippocampal neurons, without a requirement for dopamine receptors. The authors propose that dopamine activates mGluR1/5 directly and claim that mGluR1 internalization in neurons is dynamin-independent. They also provide evidence that mGluR1 recycles through a Rab11 compartment dependent on PP2B and provide evidence supporting dopamine-stimulated internalization of AMPA receptors occurring downstream of mGluR1/5 activation. While direct activation of mGluR1 by dopamine, without a requirement for known dopamine receptors, is an intriguing concept, we have major concerns about experimental design, data analysis and interpretation that undermine the conclusions claimed.

© 2026. Published by The Company of Biologists under the terms of the Creative Commons Attribution License (https://creativecommons.org/licenses/by/4.0/).

Major points:

1. The methods regarding image processing, analysis and quantification are unclear. In the "Image Acquisition and Analysis" section, the authors describe multiple thresholding steps without adequate explanation or rationale for the purpose of each step. For example, this part was particularly unclear: "To measure the surface receptors, raw images from individual experiments were maximally projected and thresholded for surface fluorescence measurement using identical values for a particular experimental condition. The thresholded areas of fluorescently labelled surface receptors were divided by the cell area, which was determined by measuring the background fluorescence using a low threshold level." (pg 33, lines 46-56). What is the purpose of these thresholding and normalization steps? Is intensity or area being measured, or both? Was this also done for the internalized receptor signal? Why are different threshold values used for different for each experimental condition? These experimental concerns are critical because they diminish confidence in a large fraction of interpretations claimed in the present study.

2. The possibility that dopamine is acting through dopamine receptors is excluded by use of 'dopamine receptor blockers'. However, the agents used (chlorpromazine hydrochloride and haloperidol) are selective antagonists of D2-like dopamine receptors. Therefore, the authors do not exclude the possibility that the effects reported in this study occur downstream of D1-like dopamine receptors, specifically D1 or D5. Hippocampal neurons are well known to produce cAMP responses to D1/5 agonists, and transcriptomic data suggest that D5 receptors are expressed at higher levels than D2-like dopamine receptors in these neurons. Without assessing potential D1/5 effects, the present pharmacological argument for dopamine acting on mGluRs, or any non-DR target, is not convincing.

3. The data suggesting that dopamine leads to activation of Gq-mediated signaling via mGluR1/5 is very weak. The mGluR1/Gαq co-immunoprecipitation in HEK293 cells is consistent with some interaction but it is not evidence for functional activation of Gq as claimed. In fact, because activation by receptors causes Gq to dissociate, the opposite result might be expected. Demonstrating functional coupling would require assaying activation of Gαq or a Gq-coupled signaling pathway downstream. One possibility is phospho-ERK assay. This is shown for hippocampal cultures, where D1/5 receptor effects are a confounding factor, but not for 293 cells. Therefore, the claimed ability of dopamine to directly activate mGluRs is not established.

4. Another potential confound is whether or how extracellular glutamate is controlled. All the effects reported can be stimulated by glutamate, and the authors provide evidence that some are blocked by a GluR1 antagonist. A simple explanation for the observed effects is that all result from an increase in extracellular Glu (such as through D1/5-cAMP increase in excitability), and this is not excluded. Was extracellular glutamate concentration controlled, and if so, how? Can the authors exclude the possibility that the observed effects result from increased glutamate?

5. The authors report dynamin-independent internalization of mGluR1 in neurons, contrasting with the dynamin-dependent internalization reported previously by others in 293 cells. This is an interesting potential mechanistic difference, but there are major caveats not addressed. The proposed dynamin-independent nature of mGluR internalization is based on dynasore or si-dynamin not blocking mGluR internalization. Beyond concern #1, about how internalization is measured, there is no positive control for the effectiveness of either manipulation. This is a significant concern because the effectiveness of dynasore is known to be reduced by off-target binding to components of serum, yet the experiments are done in B27, and because the knockdown effect of si-dynamin is partial and it is not specified which paralogues are knocked down. Neurons express three dynamins encoded by different genes, and it is not stated if the si-dynamin reagent knocks down all of them or just one. Additional information and controls are needed to establish the claim that mGluR1 internalization in neurons is dynamin-independent.

Minor points:

* In the discussion, the authors speculate that "dopamine induces internalization of group I mGluRs probably through its direct interaction with the receptor." (pg 21, lines 22-24). In my opinion, the present data do not support this claim, and making this claim would require a more incisive analysis (such as radioligand binding and direct G protein coupling analysis of isolated

© 2026. Published by The Company of Biologists under the terms of the Creative Commons Attribution License (https://creativecommons.org/licenses/by/4.0/).

mGluRs. This is an intriguing speculation but there is no evidence here to support it. Without substantially more evidence, the authors should acknowledge that whether dopamine binds directly to (and activates) mGluR1/5 remains unknown and would require additional experiments to test.

* Catalog numbers for antibodies should be included in the methods.

* Sequences for siRNAs should be provided in the methods. Which isoform(s) of clathrin heavy chain and dynamin were targeted?

Reviewer 2

SUMMARY OF THE ADVANCE MADE IN THIS PAPER AND ITS POTENTIAL SIGNIFICANCE TO THE FIELD
This article by Aruna, Kulkarni and Battacharyya provide convincing evidence that dopamine induces internalization of group I mGluRs, which is clathrin-dependent and the receptors subsequently enter recycling endosomes. The internalization of mGluR1 is PP2B dependent, which is distinct from DHPG-induced internalization. Further, they demonstrate that dopamine recruits Gï¤¡q to mGluR1. They also show that dopamine can induce the internalization of AMPARs. Overall, this is a fascinating study that provide potential insight into how dopamine might directly contribute to mGluR1-dependent plasticity relevant to learning and memory.

SUGGESTIONS TO AUTHORS

The si-Clathrin is relatively modest (~49% knockdown of protein) yet completely prevents dopamine-induced endocytosis of mGluR1. It is important that the authors control for potential off-target effects by using a different siRNA or rescuing the si-Clathrin phenotype.

Typically, the interaction between dopamine and mGluR1 is indirect via crosstalk between receptors and/or intracellular signaling. Can the authors speculate how dopamine might activate the mGluR1-dependent signaling pathways identified in this study? The use of non-competitive type-I mGluR antagonists to block the effects of dopamine is informative, but can competitive antagonism of type-I mGluRs provide additional insight?

Minor comments

The inclusion of statistics makes the main text difficult to read. Can the authors move their statistics to the figure legends or to a table?

First revision

Author response to reviewers' comments

Dear Editor,
I am happy to resubmit the paper titled “Internalization of group I metabotropic glutamate receptors and synaptic AMPA receptor endocytosis by dopamine” (MS ID#: jcs.264725). I am also submitting a point by point reply of the comments that have been made by the reviewers. We are thankful to the reviewers for their critical comments about the manuscript. Needless to say, their comments have helped us enormously to improve the quality of the manuscript. The new experiments and modifications have strengthened our conclusions enormously and thus have improved the quality of the manuscript significantly. I hope that now you will find this manuscript suitable for publication in your journal. Hope to hear a positive reply from you soon.

Response to the reviewers:

Response to the reviewer 1:

© 2026. Published by The Company of Biologists under the terms of the Creative Commons Attribution License (https://creativecommons.org/licenses/by/4.0/).

1) **Comment:** The methods regarding image processing, analysis and quantification are unclear. In the "Image Acquisition and Analysis" section, the authors describe multiple thresholding steps without adequate explanation or rationale for the purpose of each step. For example, this part was particularly unclear: "To measure the surface receptors, raw images from individual experiments were maximally projected and thresholded for surface fluorescence measurement using identical values for a particular experimental condition. The thresholded areas of fluorescently labelled surface receptors were divided by the cell area, which was determined by measuring the background fluorescence using a low threshold level." (pg 33, lines 46-56). What is the purpose of these thresholding and normalization steps? Is intensity or area being measured, or both? Was this also done for the internalized receptor signal? Why are different threshold values used for different for each experimental condition? These experimental concerns are critical because they diminish confidence in a large fraction of interpretations claimed in the present study.

   Reply: We agree with the reviewer that in a study the appropriate experimental methodologies and quantitative approaches are critical. Let us first state at the beginning that the experimental methodologies and quantitative approaches that we have taken in this study have been used by large number of labs across the world including our lab. Therefore, these methods and approaches are well established and well accepted. Space limitations in the manuscript did not allow us to explain the experimental procedures and quantitative approaches taken in this study in great detail. Since, the reviewer raised this issue let us explain them in detail. Let us first explain in detail the rationale behind using this “live cell dual antibody staining assay” and then the rationale behind the quantitation of the surface fluorescence and internalization index using threshold in ImageJ software. We studied the endocytosis and recycling of group I mGluRs using “live cell dual antibody staining” method as described in the “materials and methods” section of the manuscript. The advantage of this technique is it measures the proportion of the surface receptors that are internalized and thus variability in the surface expression of the receptor in different cells (due to the transient transfection of myc-mGluR1 or myc-mGluR5 in case of group I mGluRs and variability of endogenous AMPA receptor expression between cells in primary hippocampal neurons) does not affect the endocytosis measurement. We have normalized the endocytosis within each cell and thus variability in the surface expression of the receptor in different cells did not affect the endocytosis and recycling measurements. Similarly, for the receptor cell surface expression measurement, we have measured the surface expression of the respective receptors and normalized them with the cell area. This in cell normalization method again took care of the variability in the surface expression of the receptor in different cells. Furthermore, we have used Alexa-647 conjugated secondary antibody to visualize the internalized receptors since Alexa-647 is not detectable in the visible range, which enabled us for an unbiased blind sampling of cells in various conditions. Using this method we could reliably observe the endocytosis of group I mGluRs and AMPA receptors. We could also very reliably measure the cell surface expression of the respective receptors. We and other labs performed the endocytosis and recycling assays of group I mGluRs, AMPA receptors and other receptors and measured their cell surface expression and internalization index exactly the same way in earlier studies (Bhattacharyya et al., 2009; Biou et al., 2008; Citri et al., 2010; Citri et al., 2009; Gulia et al., 2017; Mahato et al., 2015; Ojha et al., 2022; Pandey et al., 2014; Pandey et al., 2020; Ramsakha et al., 2023; Sharma et al., 2018; Trivedi and Bhattacharyya, 2012).

   Let us now explain how quantitation of the cell surface expression and internalization index of the receptors were measured using threshold in ImageJ software. As mentioned before, these quantitations were done using the procedure that is well established and have been used by large number of labs across the world, including our lab. As mentioned in the manuscript, images from all the conditions in a particular experiment were obtained using identical parameters. All analyses were done blind using raw images and quantitation was done using ImageJ software. Raw images from each experiment were maximally projected and thresholded using equal values for different experimental conditions. Thresholding cells for quantitation separates real fluorescence

© 2026. Published by The Company of Biologists under the terms of the Creative Commons Attribution License (https://creativecommons.org/licenses/by/4.0/).

from background by creating a binary mask, typically based on pixel intensity histograms, to enable accurate measurement of intensity or area. We were careful in selecting the threshold value since over-thresholding may discard low intensity signals and under-thresholding may include background noise. We applied the same thresholding values across all comparative images to avoid bias. As mentioned in the manuscript, we measured the thresholded **area** in every case. For the cell surface localized receptor measurements, raw images from individual experiment were maximally projected and thresholded for surface fluorescence measurement using identical values for a particular experimental condition. We used two threshold values in this case. One threshold value was applied for thresholding the fluorescence of the cell surface localized receptors and the second threshold value for thresholding the background fluorescence of the cell that essentially covered the entire cell, which was measured as cell area. The area occupied by the thresholded surface florescence signal was divided by the thresholded area that covers almost the entire cell area of the cell. As mentioned before, this in cell normalization was performed so that variability of the cell surface expression of the receptor between cells does not influence the measurement of the cell surface localized receptors. These values were then normalized to the average fluorescence of control cells. For the measurement of the endocytosis index, raw images were first maximally projected. Subsequently, the surface fluorescence (cell surface localized receptors that did not endocytose upon ligand application) was thresholded using one value and internalized fluorescence (fluorescence from the internalized receptors following ligand application) was thresholded using another value. Again, we applied the same thresholding values across all comparative images to avoid bias. As mentioned in the manuscript, following that the internalization index for each cell was then calculated by dividing the value for the thresholded area contributed by the internal fluorescence with the value for the thresholded area contributed by the total fluorescence (surface + internal). These values were then normalized with respect to their controls. Again, this in cell normalization of the internalization index was performed, so that the variability of the cell surface expression of the receptor between cells does not influence the endocytosis measurement. Hope this clarifies the logic and rationale behind the protocols for performing our experiments as well as the quantitation approaches using threshold.

As mentioned before, along with large number of labs across the world, we have also used this method for studying and quantifying the cell surface expression of the receptors (group I mGluRs and AMPA receptors) and endocytosis index of these receptors in our previous studies (Bhattacharyya et al., 2009; Biou et al., 2008; Citri et al., 2010; Citri et al., 2009; Gulia et al., 2017; Mahato et al., 2015; Ojha et al., 2022; Pandey et al., 2014; Pandey et al., 2020; Ramsakha et al., 2023; Sharma et al., 2018; Trivedi and Bhattacharyya, 2012).

2) **Comment:** The possibility that dopamine is acting through dopamine receptors is excluded by use of 'dopamine receptor blockers'. However, the agents used (chlorpromazine hydrochloride and haloperidol) are selective antagonists of D2-like dopamine receptors. Therefore, the authors do not exclude the possibility that the effects reported in this study occur downstream of D1-like dopamine receptors, specifically D1 or D5. Hippocampal neurons are well known to produce cAMP responses to D1/5 agonists, and transcriptomic data suggest that D5 receptors are expressed at higher levels than D2-like dopamine receptors in these neurons. Without assessing potential D1/5 effects, the present pharmacological argument for dopamine acting on mGluRs, or any non-DR target, is not convincing.

   Reply: We would like to point out that according to the reported K_i values of chlorpromazine hydrochloride and haloperidol at D1 (D1 and D5 subtypes) and D2-like (D2, D3 and D4 subtypes) dopamine receptors, the concentrations of these drugs that we have used in our studies (10 μM chlorpromazine hydrochloride and 10 μM haloperidol) inhibits both D1-like dopamine receptors and D2-like dopamine receptors (Faedda et al., 1989). Therefore, in order to rule out the possibility that dopamine-mediated internalization of mGluR1/mGluR5 was due to the activation of dopamine receptors by dopamine, in our assays we indeed blocked both the D1-like and D2-like

© 2026. Published by The Company of Biologists under the terms of the Creative Commons Attribution License (https://creativecommons.org/licenses/by/4.0/).

dopamine receptors. Subsequently, we studied the dopamine-mediated internalization of group I mGluRs in primary hippocampal neurons.

Although in our experiments that we reported in the manuscript, we did block both D1 and D2-like dopamine receptors, but since the reviewer raised this issue, we have again performed this same experiment using chlorpromazine hydrochloride, haloperidol and a specific potent D1-like receptor blocker, SCH23390, which inhibits both D1 and D5 dopamine receptors (Bourne, 2001). Our data again suggested that the dopamine-mediated internalization of myc-mGluR1 was not inhibited in the presence of 10 µM chlorpromazine hydrochloride, 10 µM haloperidol and 10 µM SCH23390 in primary hippocampal neurons. We have shown the result below:

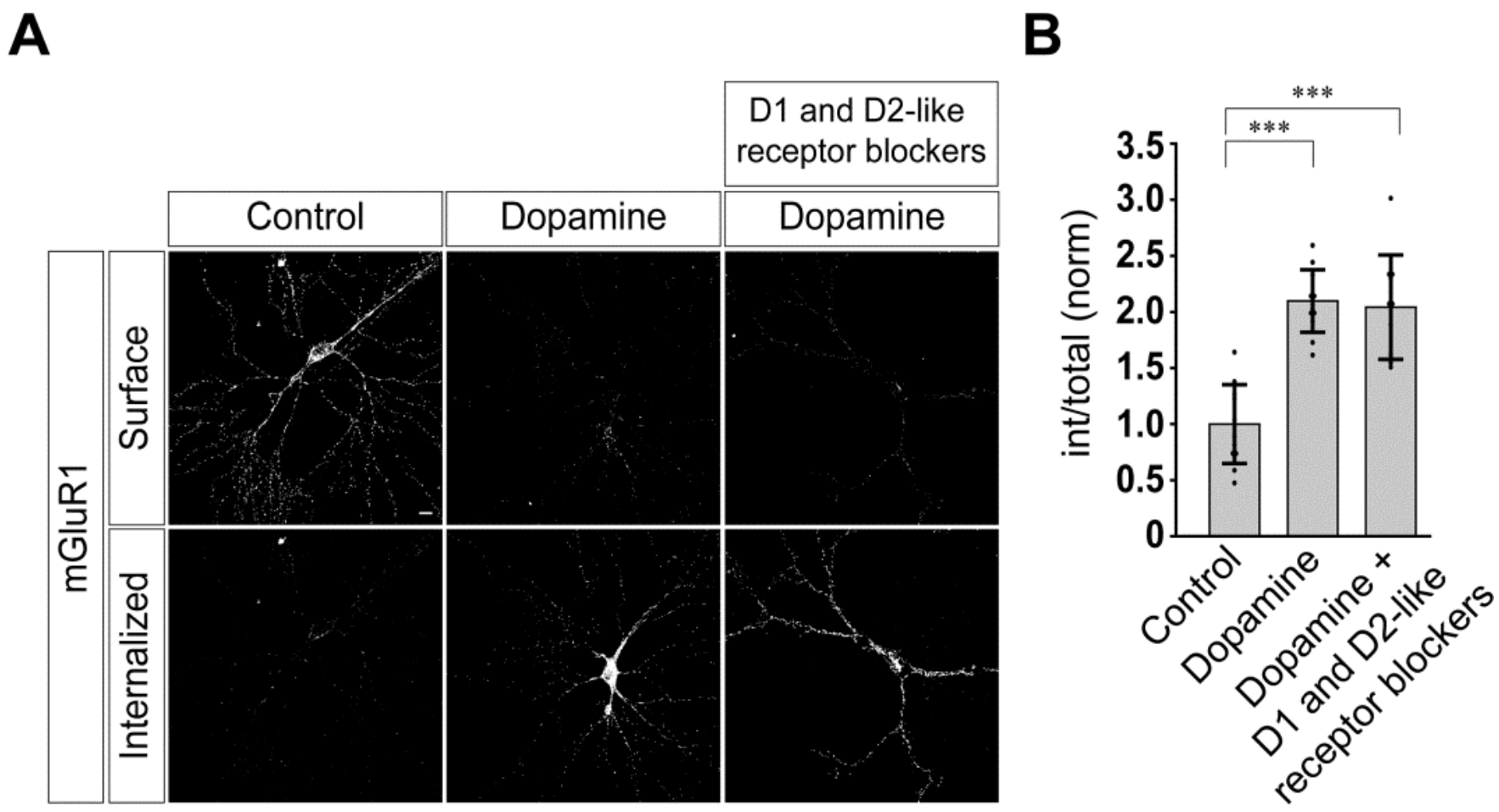

D1 and D2-like dopamine receptor blockers do not inhibit the dopamine-mediated internalization of mGluR1. (A, B) Representative images (A) and quantitation suggest that application of D1 and D2-like dopamine receptors blockers (10 µM chlorpromazine hydrochloride, 10 µM haloperidol and 10 µM SCH23390) did not inhibit the dopamine-mediated internalization of myc-mGluR1. All the results represented as means ± SD. Scale bar = 10 µm. ***, $p < 0.001$.

Therefore, together these experiments conclusively suggest that dopamine-mediated internalization of group I mGluRs was not due to the activation of dopamine receptors by dopamine.

3) **Comment**: The data suggesting that dopamine leads to activation of Gq-mediated signaling via mGluR1/5 is very weak. The mGluR1/Gαq co-immunoprecipitation in HEK293 cells is consistent with some interaction but it is not evidence for functional activation of Gq as claimed. In fact, because activation by receptors causes Gq to dissociate, the opposite result might be expected. Demonstrating functional coupling would require assaying activation of Gαq or a Gq-coupled signaling pathway downstream. One possibility is phospho-ERK assay. This is shown for hippocampal cultures, where D1/5 receptor effects are a confounding factor, but not for 293 cells. Therefore, the claimed ability of dopamine to directly activate mGluRs is not established.

   Reply: We thank the reviewer for this comment and suggestion. The purposes of these experiments were to demonstrate the physical association of mGluR1 with its cognate G-protein in our heterologous expression system HEK293 cells and to examine whether dopamine treatment alters this interaction. Our results suggest that similar to the application of the agonist R,S-DHPG, application of dopamine also leads to the increase in the interaction of the receptor with $G\alpha_q$. The reviewer mentioned that "because activation by receptors causes $G\alpha_q$ to dissociate, the opposite result might be expected". It should be noted that in the canonical GPCR signaling pathway, following ligand binding, first the receptor interacts with the G-protein involved. Subsequently,

© 2026. Published by The Company of Biologists under the terms of the Creative Commons Attribution License (https://creativecommons.org/licenses/by/4.0/).

the receptor gets dissociated with the G-protein. Following is the sequence of events in GPCR signaling:

When ligand binds to the extracellular side of the GPCR, it induces a conformational change in the seven transmembrane helices of the receptor. This conformational change opens a binding pocket for G-protein binding in the intracellular side of the receptor. In some cases, heterotrimeric G-proteins Gαβγ may be loosely associated with the receptor even before the ligand binding. Subsequent to the conformational change of the receptor, the intracellular pocket of the receptor binds the G-protein. This interaction causes a conformational change in the Gα subunit of the G-protein, which leads to the exchange of GDP for GTP. This in turn leads to the activation/inhibition of the downstream second messenger pathway. Once GTP binds to the Gα subunit, the complex becomes unstable, leading to the dissociation of the Gα-GTP subunit from Gβγ subunits as well as their dissociation from the receptor.

Therefore, in our assays when we fixed the cells at a very early time point, which was 2 min following R,S-DHPG/dopamine application, we could observe the increase in the interaction of the $G\alpha_q$ with mGluR1 upon R,S-DHPG/dopamine application. Needless to say, when we waited for longer time, i.e., we fixed the cells at a later time point following R,S-DHPG/dopamine application, we could not see this interaction, since the receptor was dissociated from the $G\alpha_q$. We have mentioned the protocol in detail in the "methods" section in our manuscript. Hope this clarifies the point.

We agree with the reviewer that increase in the interaction of the receptor with the G-protein upon ligand application is not an evidence for functional activation of the G-protein. That is why we did the assay to study the upregulation of the phosphorylation of MAPK/ERK1/2 upon application of dopamine in primary neurons. This assay suggested the activation of the G-protein and subsequent activation of the second messenger pathway upon activation of group I mGluRs by dopamine. As suggested by the reviewer, we have now performed this experiment in heterologous HEK293 cells, where D1-like or D2-like dopamine receptors effects are not a confounding factor. Our data suggest that in myc-mGluR1 expressing HEK293 cells also application of dopamine led to the upregulation of the phosphorylation of MAPK/ERK1/2. Importantly, dopamine could not increase the phosphorylation of MAPK/ERK1/2 in presence of the mGluR1 antagonist or $G\alpha_q$ inhibitor. We have included this data in our revised manuscript (Fig. 5E, F). Together these results strongly suggest that similar to the agonist R,S-DHPG, dopamine also increases $G\alpha_q$ activity mediated by group I mGluRs and subsequent activation of the MAPK/ERK1/2 pathway in both heterologous HEK293 cells and primary neurons.

4) **Comment:** Another potential confound is whether or how extracellular glutamate is controlled. All the effects reported can be stimulated by glutamate, and the authors provide evidence that some are blocked by a GluR1 antagonist. A simple explanation for the observed effects is that all result from an increase in extracellular Glu (such as through D1/5-cAMP increase in excitability), and this is not excluded. Was extracellular glutamate concentration controlled, and if so, how? Can the authors exclude the possibility that the observed effects result from increased glutamate?

   Reply: We would like to emphasize that all our experiments were performed in glutamate free and B-27 free media. In HEK293 cells experiments were performed in plain DMEM (without serum and glutamate) and in primary hippocampal neurons in plain neurobasal media (without glutamate and B-27). We have now clearly mentioned it in the revised manuscript. Primary neurons were cultured in B-27 containing neurobasal media. They were washed multiple times before starting the experiments and all experiments were performed in plain neurobasal media (glutamate free and B-27 free). The extracellular media did not have glutamate or B-27 during the entire experiment. We have used this protocol extensively to study the R,S-DHPG mediated trafficking of group I mGluRs in our earlier studies (Gulia et al., 2017; Ojha et al., 2022; Pandey et al., 2020; Ramsakha et al., 2023; Sharma et al., 2018). On top of that, as mentioned before, we have repeated these experiments in presence of 10 μM

© 2026. Published by The Company of Biologists under the terms of the Creative Commons Attribution License (https://creativecommons.org/licenses/by/4.0/).

chlorpromazine hydrochloride and 10 µM haloperidol which inhibit both D1-like dopamine receptors (D1 and D5 subtypes) and D2-like dopamine receptors (D2, D3 and D4 subtypes). Together, it strongly nullifies the concern of the reviewer about the contribution of the extracellular glutamate.

Although in our experiments the possibility of presence of extracellular glutamate does not exists, since the reviewer raised this issue, we have again performed this experiment in presence of the glutamate release blocker Riluzole (30 µM) (Doble, 1996; Kretschmer et al., 1998). Our data again suggested that dopamine-mediated internalization of myc-mGluR1 was not inhibited in the presence of 30 µM Riluzole. We have shown the data below:

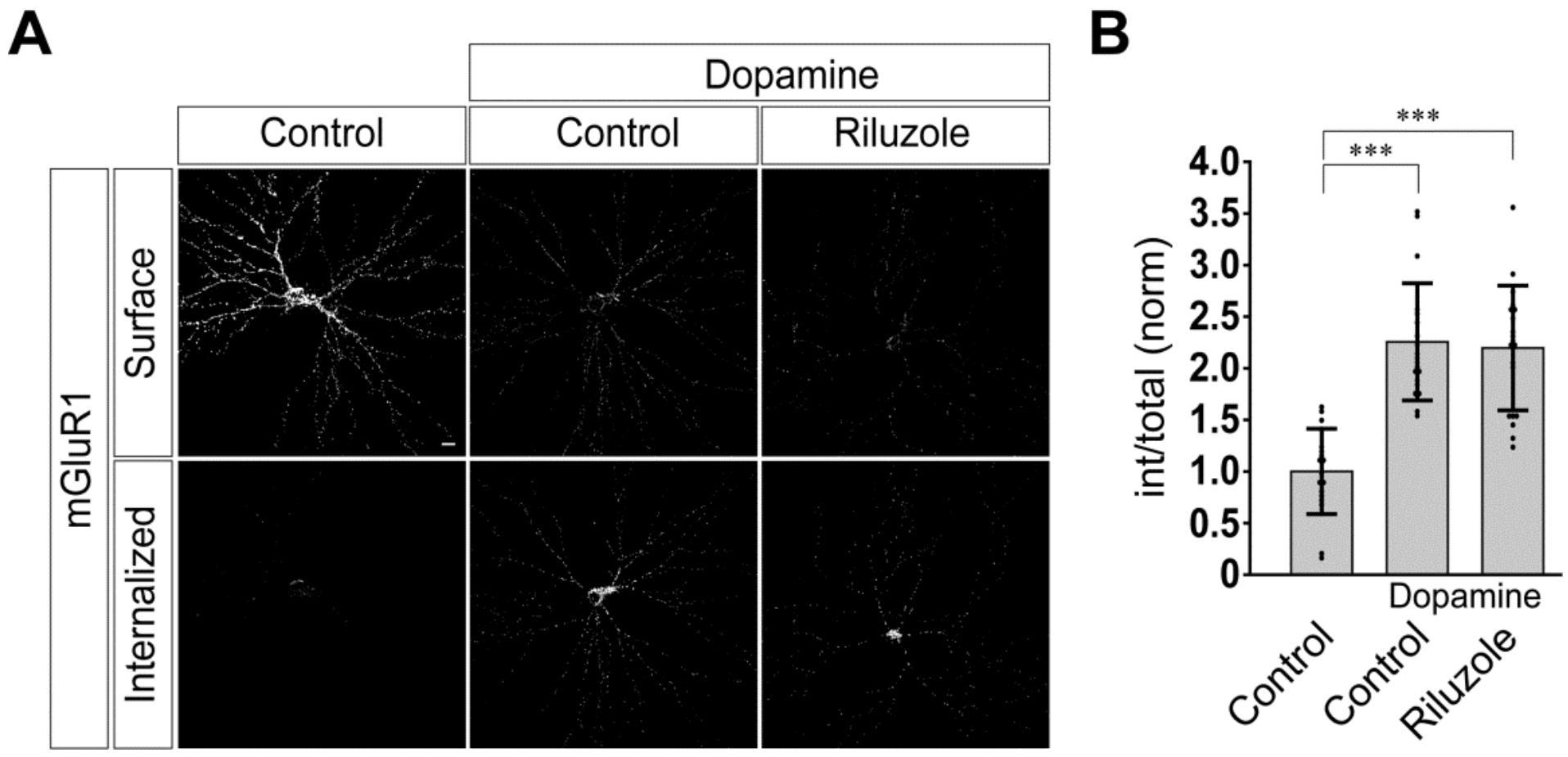

Dopamine-mediated internalization of mGluR1 in presence of the glutamate release blocker. (A, B) Representative images (A) and quantitation (B) suggest that glutamate release inhibitor; Riluzole did not inhibit the dopamine-mediated internalization of myc-mGluR1. All the results represented as means ± SD. Scale bar = 10 µm. ***, $p < 0.001$.

Together all these facts and results rule out the possibility of any contribution of extracellular glutamate in the dopamine-mediated internalization of mGluR1.

5) **Comment:** The authors report dynamin-independent internalization of mGluR1 in neurons, contrasting with the dynamin-dependent internalization reported previously by others in 293 cells. This is an interesting potential mechanistic difference, but there are major caveats not addressed. The proposed dynamin-independent nature of mGluR internalization is based on dynasore or si-dynamin not blocking mGluR internalization. Beyond concern #1, about how internalization is measured, there is no positive control for the effectiveness of either manipulation. This is a significant concern because the effectiveness of dynasore is known to be reduced by off-target binding to components of serum, yet the experiments are done in B27, and because the knockdown effect of si-dynamin is partial and it is not specified which paralogues are knocked down. Neurons express three dynamins encoded by different genes, and it is not stated if the si-dynamin reagent knocks down all of them or just one. Additional information and controls are needed to establish the claim that mGluR1 internalization in neurons is dynamin-independent.

Reply: We agree with the reviewer that the dynamin-independent mechanism of dopamine-mediated internalization of mGluR1 is a very interesting observation. As stated in the manuscript, it has been reported that the agonist-mediated internalization of mGluR1 is dynamin-dependent (Mundell et al., 2001). Therefore, dynamin-independent internalization of the receptor upon application of dopamine is an interesting mechanistic difference. We would like to clarify again that all our experiments have been performed in glutamate free and B-27 free plain neurobasal media (not in the presence of B-27 as mentioned by the reviewer). We have now

© 2026. Published by The Company of Biologists under the terms of the Creative Commons Attribution License (https://creativecommons.org/licenses/by/4.0/).

mentioned it clearly in the revised manuscript. The reviewer advised us to have a positive control for dynasore and si-dynamin. We had the positive control for si-dynamin in the manuscript (Fig. 4I, J). We studied the agonist (R,S-DHPG)-mediated internalization of myc-mGluR1 upon acute knockdown of the endogenous dynamin in primary hippocampal neurons. As pointed out earlier, the agonist-mediated internalization of mGluR1 has been reported to be dynamin-dependent. Expectedly, knockdown of the endogenous dynamin led to the complete inhibition of the 100 µM R,S-DHPG-mediated internalization of myc-mGluR1 (Fig. 4I, J). Thus, this experiment served as a positive control for si-dynamin. We have knocked down dynamin-1 through our siRNA and we have now mentioned that in the revised manuscript. We knocked down dynamin-1 since this isoform of dynamin is almost exclusively expressed in the central nervous system (Ferguson et al., 2007; Sontag et al., 1994).

As suggested by the reviewer, we have now performed the positive control experiment to show that dynasore indeed inhibited the endogenous dynamin in primary hippocampal neurons, by performing the R,S-DHPG-mediated internalization of myc-mGluR1 in presence of dynasore. Our data suggested that application of 80 µM dynasore (same concentration that was used in the dopamine-mediated myc-mGluR1 internalization assay) completely inhibited the 100 µM R,S-DHPG-mediated internalization of myc-mGluR1 in primary hippocampal neurons (Fig. S2A, B). We have included this result in the “supplementary information”. Therefore, we have now presented experiments that served as positive controls for both dynasore and si-dynamin and all these experiments were performed in glutamate free, B-27 free plain neurobasal media. Together, these results strongly suggest that dopamine-mediated internalization of mGluR1 is dynamin-independent.

6) **Comment:** In the discussion, the authors speculate that "dopamine induces internalization of group I mGluRs probably through its direct interaction with the receptor." (pg 21, lines 22-24). In my opinion, the present data do not support this claim, and making this claim would require a more incisive analysis (such as radioligand binding and direct G protein coupling analysis of isolated mGluRs. This is an intriguing speculation but there is no evidence here to support it. Without substantially more evidence, the authors should acknowledge that whether dopamine binds directly to (and activates) mGluR1/5 remains unknown and would require additional experiments to test.

Reply: We completely agree with the reviewer. That is why we added the word “probably” in that sentence. As suggested by the reviewer, we have now added the following sentence in the “discussion” section of the revised manuscript: “However, it is to be noted that in order to know whether dopamine binds directly to the group I mGluRs and activates them directly, more experiments need to be performed in future.”

7) **Comment:** Catalogue numbers for antibodies should be included in the methods.

Reply: We have included the catalogue numbers of antibodies used in this study in the “materials” section of the revised manuscript.

8) **Comment:** Sequences for siRNAs should be provided in the methods. Which isoform(s) of clathrin heavy chain and dynamin were targeted?

Reply: We have now provided the sequences of the siRNAs and shRNAs in the supplementary information of the revised manuscript. We have also mentioned in the text of the revised manuscript that knockdown of the clathrin heavy chain and dynamin-1 isoform were performed in our studies. We knocked down dynamin-1 since this isoform of dynamin is almost exclusively expressed in the central nervous system (Ferguson et al., 2007; Sontag et al., 1994).

<u>Response to the reviewer 2:</u>

1) **Comment:** This article by Aruna, Kulkarni and Bhattacharyya provide convincing evidence that dopamine induces internalization of group I mGluRs, which is clathrin-

© 2026. Published by The Company of Biologists under the terms of the Creative Commons Attribution License (https://creativecommons.org/licenses/by/4.0/).

dependent and the receptors subsequently enter recycling endosomes. The internalization of mGluR1 is PP2B dependent, which is distinct from DHPG-induced internalization. Further, they demonstrate that dopamine recruits Gαq to mGluR1. They also show that dopamine can induce the internalization of AMPARs. Overall, this is a fascinating study that provide potential insight into how dopamine might directly contribute to mGluR1-dependent plasticity relevant to learning and memory.

Reply: We thank the reviewer for appreciating this study.

2) **Comment:** The si-Clathrin is relatively modest (~49% knockdown of protein) yet completely prevents dopamine-induced endocytosis of mGluR1. It is important that the authors control for potential off-target effects by using a different siRNA or rescuing the si-Clathrin phenotype.

   Reply: We thank the reviewer for this suggestion. We have now performed the same experiment by knocking down the endogenous clathrin by shRNA. We have cloned a shRNA against endogenous clathrin in a multi-promoter vector, under the H1 promoter. We have used this vector in our previous studies (Ojha et al., 2022; Pandey et al., 2020; Sharma et al., 2018). The same shRNA has been reported to effectively knockdown the endogenous clathrin in primary hippocampal neurons (Watanabe et al., 2014). The detailed experimental protocol has been described in the "methods" section of our revised manuscript. Our data suggest that acute knockdown of the endogenous clathrin by shRNA also inhibited the dopamine-mediated internalization of myc-mGluR1 in primary hippocampal neurons (Fig. S3A, B). We have included this result in the "supplementary information". Thus, knocking down the endogenous clathrin by two different means, viz. by siRNA and shRNA gave us the same result, i.e., inhibition of the dopamine-mediated internalization of myc-mGluR1 in primary hippocampal neurons.

3) **Comment:** Typically, the interaction between dopamine and mGluR1 is indirect via crosstalk between receptors and/or intracellular signaling. Can the authors speculate how dopamine might activate the mGluR1-dependent signaling pathways identified in this study? The use of non-competitive type-I mGluR antagonists to block the effects of dopamine is informative, but can competitive antagonism of type-I mGluRs provide additional insight?

   Reply: We thank the reviewer for raising this point. Both the non-competitive antagonists of the receptors that we have used in this study (JNJ16259685 for mGluR1 and MTEP for mGluR5) bind to an allosteric site within the seven transmembrane region of the receptor. This binding site is distinct from the orthosteric glutamate/R,S-DHPG binding region of the receptor. Binding of these non-competitive antagonists to the receptor might results in a conformational change in the receptor so that either the ligand can no longer bind effectively with the receptor or can't fully activate the receptor. This hypothesis needs to be experimentally investigated in future. We have incorporated this hypothesis in the "discussion" section of the revised manuscript. We have also seen that the competitive antagonist of mGluR1 did not inhibit the dopamine-mediated internalization of myc-mGluR1 (data not shown), suggesting again that dopamine probably does not bind to the same region where glutamate/R,S-DHPG binds. As stated in the manuscript, the binding site of dopamine with mGluR1/mGluR5 needs to be determined in future.

4) **Comment:** The inclusion of statistics makes the main text difficult to read. Can the authors move their statistics to the figure legends or to a table?

   Reply: As suggested by the reviewer, we have moved the statistical values to the respective figure legends in the revised manuscript.

References

© 2026. Published by The Company of Biologists under the terms of the Creative Commons Attribution License (https://creativecommons.org/licenses/by/4.0/).

Bhattacharyya, S., Biou, V., Xu, W., Schluter, O. and Malenka, R. C. (2009). A critical role for PSD-95/AKAP interactions in endocytosis of synaptic AMPA receptors. *Nat Neurosci* **12**, 172-81.

Biou, V., Bhattacharyya, S. and Malenka, R. C. (2008). Endocytosis and recycling of AMPA receptors lacking GluR2/3. *Proc Natl Acad Sci U S A* **105**, 1038-43.

Bourne, J. A. (2001). SCH 23390: the first selective dopamine D1-like receptor antagonist. *CNS Drug Rev* **7**, 399-414.

Citri, A., Bhattacharyya, S., Ma, C., Morishita, W., Fang, S., Rizo, J. and Malenka, R. C. (2010). Calcium binding to PICK1 is essential for the intracellular retention of AMPA receptors underlying long-term depression. *J Neurosci* 30, 16437-52.

Citri, A., Soler-Llavina, G., Bhattacharyya, S. and Malenka, R. C. (2009). N-methyl-D-aspartate receptor- and metabotropic glutamate receptor-dependent long-term depression are differentially regulated by the ubiquitin-proteasome system. *Eur J Neurosci* **30**, 1443-50.

Doble, A. (1996). The pharmacology and mechanism of action of riluzole. *Neurology* **47**, S233-41.

Faedda, G., Kula, N. S. and Baldessarini, R. J. (1989). Pharmacology of binding of 3H-SCH-23390 to D-1 dopaminergic receptor sites in rat striatal tissue. *Biochem Pharmacol* **38**, 473-80.

Ferguson, S. M., Brasnjo, G., Hayashi, M., Wolfel, M., Collesi, C., Giovedi, S., Raimondi, A., Gong, L. W., Ariel, P., Paradise, S. et al. (2007). A selective activity-dependent requirement for dynamin 1 in synaptic vesicle endocytosis. *Science* **316**, 570-4.

Gulia, R., Sharma, R. and Bhattacharyya, S. (2017). A Critical Role for Ubiquitination in the Endocytosis of Glutamate Receptors. *J Biol Chem* **292**, 1426-1437.

Kretschmer, B. D., Kratzer, U. and Schmidt, W. J. (1998). Riluzole, a glutamate release inhibitor, and motor behavior. *Naunyn Schmiedebergs Arch Pharmacol* **358**, 181-90.

Mahato, P. K., Pandey, S. and Bhattacharyya, S. (2015). Differential effects of protein phosphatases in the recycling of metabotropic glutamate receptor 5. *Neuroscience* **306**, 138-50.

Mundell, S. J., Matharu, A. L., Pula, G., Roberts, P. J. and Kelly, E. (2001). Agonist-induced internalization of the metabotropic glutamate receptor 1a is arrestin- and dynamin-dependent. *J Neurochem* **78**, 546-51.

Ojha, P., Pal, S. and Bhattacharyya, S. (2022). Regulation of Metabotropic Glutamate Receptor Internalization and Synaptic AMPA Receptor Endocytosis by the Postsynaptic Protein Norbin. *J Neurosci* **42**, 731-748.

Pandey, S., Mahato, P. K. and Bhattacharyya, S. (2014). Metabotropic glutamate receptor 1 recycles to the cell surface in protein phosphatase 2A-dependent manner in non-neuronal and neuronal cell lines. *J Neurochem* **131**, 602-14.

Pandey, S., Ramsakha, N., Sharma, R., Gulia, R., Ojha, P., Lu, W. and Bhattacharyya, S. (2020). The post-synaptic scaffolding protein tamalin regulates ligand-mediated trafficking of metabotropic glutamate receptors. *J Biol Chem* 295, 8575-8588.

Ramsakha, N., Ojha, P., Pal, S., Routh, S., Citri, A. and Bhattacharyya, S. (2023). A vital role for PICK1 in the differential regulation of metabotropic glutamate receptor internalization and synaptic AMPA receptor endocytosis. *J Biol Chem* **299**, 104837.

Sharma, R., Gulia, R. and Bhattacharyya, S. (2018). A Critical Role for Sorting Nexin 1 in the Trafficking of Metabotropic Glutamate Receptors. *J Neurosci* **38**, 8605-8620.

Sontag, J. M., Fykse, E. M., Ushkaryov, Y., Liu, J. P., Robinson, P. J. and Sudhof, T. C. (1994). Differential expression and regulation of multiple dynamins. *J Biol Chem* 269, 4547-54.

Trivedi, R. R. and Bhattacharyya, S. (2012). Constitutive internalization and recycling of metabotropic glutamate receptor 5 (mGluR5). *Biochem Biophys Res Commun* **427**, 185-90.

Watanabe, S., Trimbuch, T., Camacho-Perez, M., Rost, B. R., Brokowski, B., Sohl-Kielczynski, B., Felies, A., Davis, M. W., Rosenmund, C. and Jorgensen, E. M. (2014). Clathrin regenerates synaptic vesicles from endosomes. *Nature* **515**, 228-33.

Second decision letter

MS ID#: jcs.264725R1

© 2026. Published by The Company of Biologists under the terms of the Creative Commons Attribution License (https://creativecommons.org/licenses/by/4.0/).

MS Title: Metabotropic glutamate receptor internalization and synaptic AMPA receptor endocytosis by dopamine

Authors: K Aruna; Mitra Kulkarni; Samarjit Bhattacharyya
Article Type: Research Article

Dear Dr Bhattacharyya,

I am happy to tell you that your manuscript has been accepted for publication in Journal of Cell Science, pending standard publication integrity checks.

© 2026. Published by The Company of Biologists under the terms of the Creative Commons Attribution License (https://creativecommons.org/licenses/by/4.0/).
